# Implicit quiescent soliton perturbation in optical metamaterials with complex Ginzburg–Landau equation having nonlinear chromatic dispersion and Kudryashov’s forms of self–phase modulation structures by lie symmetry

**DOI:** 10.1016/j.mex.2026.103842

**Published:** 2026-02-20

**Authors:** Abdullahi Rashid Adem, Ahmed H. Arnous, Houria Triki, Oswaldo González–Gaxiola, Lina S. Calucag, Anjan Biswas

**Affiliations:** a Department of Mathematical Sciences, University of South Africa, UNISA 0003, South Africa; b Department of Mathematical Sciences, Saveetha School of Engineering, SIMATS, Chennai 602105, Tamilnadu, India; c Research Center of Applied Mathematics, Khazar University, Baku, AZ 1096, Azerbaijan; d Radiation Physics Laboratory, Department of Physics, Faculty of Sciences, Badji Mokhtar University, P. O. Box 12, 23000, Annaba, Algeria; e Applied Mathematics and Systems Department, Universidad Autónoma Metropolitana–Cuajimalpa, Vasco de Quiroga 4871, Mexico City 05348, Mexico; f Department of Mathematics and Science, University of Technology Bahrain, Kingdom of Bahrain; g Department of Mathematics and Physics, Grambling State University, Grambling, LA 71245–2715, USA; h Department of Physics and Electronics, Khazar University, Baku, AZ 1096, Azerbaijan; i Department of Mathematics and Applied Mathematics, Sefako Makgatho Health Sciences University, Medunsa 0204, South Africa; j Applied Science Research Center, Applied Science Private University, Amman 11937, Jordan

**Keywords:** Quiescent solitons, Metamaterials, Chromatic dispersion

## Abstract

The paper explores the retrieval of quiescent perturbed solitons in optical metamaterials characterized by nonlinear chromatic dispersion and Kudryashov’s forms of self-phase modulation structures. The foundational model utilized is the complex Ginzburg-Landau equation. The novelty is methodological, as we use Lie symmetry reduction to transform the stationary traveling-wave system into an invariant first-order form, yielding unified quadratures and explicit existence constraints for quiescent solitons across all Kudryashov SPM variants without imposing ad hoc trial functions. A successful implementation of Lie symmetry analysis has been conducted for this retrieval process. In particular, the symmetry-based reduction produces algebraic admissibility conditions that provide a reproducible parameter-screening rule for localized profiles. Additionally, both linear and generalized temporal evolutions are considered. Representative parameter sweeps illustrate how the Kudryashov coefficients and dispersive parameters control the soliton amplitude and width within the admissible windows predicted by the theory. These analyses offer helpful details about the stability and dynamics of the solitons, in the sense of identifying regimes where stationary localization is supported by the reduced dynamical system and where it is precluded by violation of the existence conditions, thereby highlighting potential applications in advanced optical communication systems and providing explicit design-side parameter limits that can help avoid operating regimes associated with pulse degradation.

• The study models quiescent soliton perturbation in optical metamaterials using a perturbed complex Ginzburg–Landau framework that incorporates nonlinear chromatic dispersion and Kudryashov-type non-Kerr self-phase modulation structures.
• Lie symmetry is applied to retrieve quiescent soliton profiles in implicit form, producing integral-form solutions together with constraint conditions on the governing parameters.
• The analysis considers both linear and generalized temporal evolutions and treats multiple Kudryashov self-phase modulation variants, clarifying how evolution choice and SPM structure influence the resulting soliton behavior and potential optical applications.

## Graphical abstract

**Figure:**
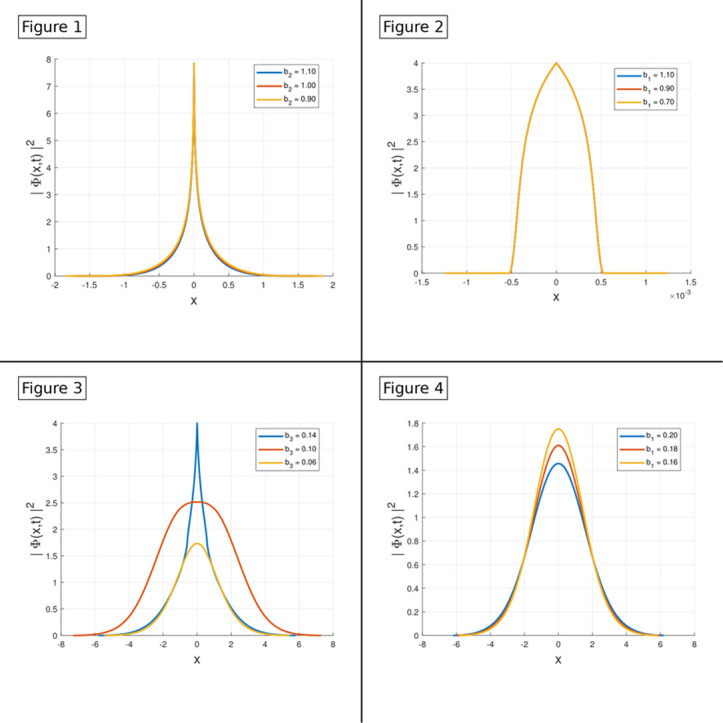

**Specifications table**

**Table:** 

**Subject area**	Mathematics and Statistics
**More specific subject area**	Mathematical Physics
**Name of your method**	Lie symmetry retrieval of implicit quiescent solitons
**Name and reference of original method**	Lie symmetry analysis (classical group invariance) ***A. R. Adem, Y. Yildirim,* L. *Moraru, O. González–Gaxiola & A. Biswas. “Implicit quiescent optical soliton perturbation having nonlinear chromatic dispersion and generalized temporal evolution with Kudryashov’s forms of self-phase modulation structure by Lie symmetry". Afrika Matematica. Volume 36, Issue 4, Article 173. (2025).***
**Resource availability**	Mathematica

## Background

The study of quiescent optical solitons has recently gained popularity [[1], [2], [3], [4], [5], [6], [7], [8], [9], [10], [11], [12], [13], [14], [15], [16], [17], [18], [19], [20]]. These solitons result from a lost balance between chromatic dispersion (CD) and self-phase modulation (SPM). Several factors can contribute to this loss of balance. One possibility is that the CD becomes nonlinear during the transmission of the solitons. This imbalance may arise from random variations in fiber diameter, random injection of initial pulses at the fiber’s entry point, and other similar factors. These conditions create quiescent optical solitons, which stop the transmission of these pulses when they are sent underground or underwater. This stalling effect can significantly degrade signal quality and limit the effective range of optical communication systems. To mitigate these issues, researchers are exploring advanced techniques such as adaptive pulse shaping and the use of specially designed fibers that can better accommodate the nonlinear effects.

The current paper investigates the formation of quiescent solitons in a particular type of waveguide, known as optical metamaterials, while taking into account Hamiltonian-type perturbation terms. The primary framework for this study is the complex Ginzburg-Landau equation (CGLE). It is noteworthy that the phenomenon of such solitons in optical fibers has been explored recently [1]. This paper discusses six structures of self-phase modulation (SPM) that were proposed by Kudryashov over the years. The effects of temporal evolution are analyzed in both linear and generalized forms. The integrability of the governing model is investigated through the application of Lie symmetry, which leads to the identification of implicit quiescent optical solitons. The methodology is outlined in detail, and the findings are presented in the remainder of the paper following a thorough introduction to the governing model. The results emphasize the value of these solitons in various optical applications, demonstrating their robustness under different perturbations. Additionally, future research directions are suggested to explore the potential of these solutions in nonlinear photonics.

In standard weakly nonlinear optical fibers, chromatic dispersion is primarily a linear material property determined by the frequency dependence of the refractive index, while nonlinear effects enter mainly through intensity-dependent phase modulation. In optical metamaterials, however, the effective dispersion can become nonlinear because the constitutive response is engineered through subwavelength resonant inclusions such as plasmonic elements, split-ring resonators, or multilayer composites, and the resulting effective permittivity and permeability are strongly frequency-dependent and field-dependent. In this setting, the group-velocity dispersion experienced by a pulse is not only sensitive to frequency detuning but also to the instantaneous intensity through resonant nonlinear polarization, local-field enhancement inside the inclusions, and intensity-dependent shifts of the effective resonance driven by Kerr-type and non-Kerr mechanisms. These effects produce an intensity-dependent dispersive phase, commonly referred to as nonlinear chromatic dispersion, which appears in envelope models as higher-order derivative terms whose coefficients depend on the local envelope amplitude.

Optical metamaterials are particularly susceptible to nonlinear chromatic dispersion for two reasons. First, their dispersion is typically much steeper than in homogeneous media because it is dominated by engineered resonances. Steep dispersion amplifies the impact of any intensity-induced resonance shift on the group delay and pulse broadening. Second, metamaterials exhibit strong local-field enhancement in the unit cell, so moderate input powers can generate large effective intensities at the inclusion scale, magnifying nonlinear corrections to both the refractive index and the dispersive response. Consequently, envelope descriptions for metamaterials often require dispersion terms beyond the classical linear group-velocity dispersion operator, including amplitude-dependent and gradient-dependent dispersive contributions, to accurately capture pulse evolution and the formation of quiescent localized structures.

Quiescent solitons correspond to stationary localized envelopes supported by a balance between dispersion, self-phase modulation, and, when present, weak nonconservative corrections. In the present model, nonlinear chromatic dispersion modifies the dispersive side of this balance in an intensity-dependent manner, which shifts the existence window for stationary localization and motivates the explicit admissibility conditions derived below.

## Implemented Methodology

This study will be split into two sections that come with linear temporal evolution and generalized temporal evolution. The details of the derivation are enumerated in the subsequent couple of subsections. These subsections will offer an in-depth review of the methods used to analyze each type of evolution, highlighting the key differences and implications for practical applications.

### Linear temporal evolution

The dimensionless form of the governing nonlinear Schrödinger equation (NLSE) in optical metamaterials that exhibit nonlinear CD and adhere to a non-Kerr law of self-phase modulation (SPM) is structured as follows:

$$i\Phi_{t}+a{\left({\left|\Phi \right|}^{n}\Phi \right)}_{xx}+F\left({\left|\Phi \right|}^{2}\right)\Phi$$

(1) $$=\alpha \frac{{\left|\Phi_{x}\right|}^{2}}{\Phi^{*}}+\frac{\beta }{4{\left|\Phi \right|}^{2}\Phi^{*}}\left[2{\left|\Phi \right|}^{2}{\left({\left|\Phi \right|}^{2}\right)}_{xx}-{\left\{{\left({\left|\Phi \right|}^{2}\right)}_{x}\right\}}^{2}\right]+\gamma \Phi +\sigma_{1}{\left({\left|\Phi \right|}^{2m}\Phi \right)}_{xx}+\sigma_{2}{\left|\Phi \right|}^{2m}\Phi_{xx}+\sigma_{3}{\left|\Phi \right|}^{2m}\Phi_{xx}^{*}+i\left[\lambda {\left({\left|\Phi \right|}^{2m}\Phi \right)}_{x}+\theta_{1}{\left({\left|\Phi \right|}^{2m}\right)}_{x}\Phi +\theta_{2}{\left|\Phi \right|}^{2m}\Phi_{x}\right]$$

In (1), Φ(x,t) signifies the wave amplitude and is a complex-valued function. The independent variables are x and t, denoting the spatial and temporal variables, respectively. The initial term represents the linear temporal variation, with its coefficient being $i=\sqrt{-1}$. The second term, with its coefficient being a, is the nonlinear CD, where the nonlinearity factor is dictated by the exponent n. When n=0, one collapses to linear CD. The third term is from SPM, where the functional F accounts for the nonlinear structure of intensity-dependent refractive index change. The right-hand side comes from the metamaterials that led to the formation of those expressions [13]. Also, the parameter m on the right-hand side accounts for arbitrary light intensity. On the right-hand side, the coefficients $\sigma_{j}$ for j=1,2,3 are real-valued constants. These constants are important because they influence the behavior of the system under various conditions. Understanding their influence can lead to significant advancements in the design and application of metamaterials in optical technologies.

In the context of nonlinear CD, Eq. (1) does not accommodate mobile solitons until n=0 [2]. Consequently, the quiescent optical solitons governed by (1) are considered to be of the following form:

(2) $$\Phi \left(x,t\right)=g\left(x\right)e^{i\omega t},$$

In this context, g denotes the amplitude component of the soliton, whereas the second factor signifies the phase component, with ω denoting the wave number.

This section will analyze Eq. (1) with linear temporal development for six variants of SPM structures as outlined by Kudryashov. By substituting (2) in (1) and separating the results into real and imaginary components, we arrive at the following equations

$$a\left(n+1\right)g^{n}\left(x\right)\left[g\left(x\right)g^{\prime \prime }\left(x\right)+n{\left\{g^{\prime }\left(x\right)\right\}}^{2}\right]-\left[\gamma +\omega -F\left\{g^{2}\left(x\right)\right\}\right]g^{2}\left(x\right)$$

$$-\left\{\left(2m+1\right)\sigma_{1}+\sigma_{2}-\sigma_{3}\right\}g^{2m+1}\left(x\right)g^{\prime \prime }\left(x\right)$$

(3) $$-2m\left(2m+1\right)\sigma_{1}g^{2m}\left(x\right){\left\{g^{\prime }\left(x\right)\right\}}^{2}-\alpha {\left\{g^{\prime }\left(x\right)\right\}}^{2}-\beta g\left(x\right)g^{\prime \prime }\left(x\right)=0,$$

and

(4) $$\left(2m+1\right)\lambda +2m\theta_{1}+\theta_{2}=0,$$

respectively.

The real parameters λ, $\theta_{1}$, and $\theta_{2}$ multiply the gradient-type terms that enter the imaginary (nonconservative) channel of the governing model, and therefore represent nonconservative, intensity-dependent transport/steepening effects in the metamaterial envelope dynamics (for example, nonlinear advection of the wave packet and self-steepening-like corrections). We use the term “perturbation” here in the model-extension sense relative to the conservative dispersion–SPM core, without assuming that λ, $\theta_{1}$, and $\theta_{2}$ are asymptotically small unless stated explicitly.

Upon substituting the quiescent ansatz (2) and separating real and imaginary parts, the imaginary part reduces to an algebraic compatibility condition on these transport coefficients. Thus, Eq. (4) is a solvability (consistency) condition required for the existence of stationary (quiescent) profiles under the chosen ansatz. It should not be interpreted as a universal material constraint: if Eq. (4) is not satisfied, the governing PDE may still admit solutions, but the quiescent reduction used here is no longer consistent and one must instead allow a more general traveling/phase-modulated ansatz.

By virtue of (4) Eq. (1) modifies to

$$i\Phi_{t}+a{\left({\left|\Phi \right|}^{n}\Phi \right)}_{xx}+F\left({\left|\Phi \right|}^{2}\right)\Phi$$

$$=\alpha \frac{{\left|\Phi_{x}\right|}^{2}}{\Phi^{*}}+\frac{\beta }{4{\left|\Phi \right|}^{2}\Phi^{*}}\left[2{\left|\Phi \right|}^{2}{\left({\left|\Phi \right|}^{2}\right)}_{xx}-{\left\{{\left({\left|\Phi \right|}^{2}\right)}_{x}\right\}}^{2}\right]+\gamma \Phi +\sigma_{1}{\left({\left|\Phi \right|}^{2m}\Phi \right)}_{xx}+$$

(5) $$\sigma_{2}{\left|\Phi \right|}^{2m}\Phi_{xx}+\sigma_{3}{\left|\Phi \right|}^{2m}\Phi_{xx}^{*}+i\left[\begin{array}{c} \lambda {\left({\left|\Phi \right|}^{2m}\Phi \right)}_{x}+\theta_{1}{\left({\left|\Phi \right|}^{2m}\right)}_{x}\Phi -\\ \left\{2m\theta_{1}+\left(2m+1\right)\lambda \right\}{\left|\Phi \right|}^{2m}\Phi_{x} \end{array}\right]$$

The subsequent subsections analyze the model for Kudryashov’s proposed six forms of nonlinear SPM structures.

Let

$s=|\Phi {|}^{2},$ so that the SPM contribution in the governing model is always of the form F(s)Φ.

Form-I

(a) $$F_{1}\left(s\right)=\frac{b_{1}}{s^{m}}+\frac{b_{2}}{s^{m/2}}+b_{3}\;s^{m/2}+b_{4}\;s^{m},$$

where $b_{j}\neq 0$(1≤j≤4) are real constants.

Form-II

(b) $$F_{2}\left(s\right)=\frac{b_{1}}{s^{2m}}+\frac{b_{2}}{s^{3m/2}}+\frac{b_{3}}{s^{m}}+\frac{b_{4}}{s^{m/2}}+b_{5}\;s^{m/2}+b_{6}\;s^{m}+b_{7}\;s^{3m/2}+b_{8}\;s^{2m},$$

where $b_{j}\neq 0$(1≤j≤8) are real constants.

Form-III

(c) $$F_{3}\left(s\right)=b_{1}\;s^{m/2}+b_{2}\;s^{m}+b_{3}\;{\left(s^{m/2}\right)}_{xx},$$

where $b_{j}\neq 0$(1≤j≤3) are real constants and ${\left(\cdot \right)}_{xx}$ denotes the second derivative with respect to x.

Form-IV

(d) $$F_{4}\left(s\right)=b_{1}\;s^{m/2}+b_{2}\;s^{m}+b_{3}\;s^{3m/2}+b_{4}\;s^{2m}+b_{5}\;s^{5m/2}+b_{6}\;s^{3m},$$

where $b_{j}\neq 0$(1≤j≤6) are real constants.

Form-V

(e) $$F_{5}\left(s\right)=b_{1}\;s^{m/2}+b_{2}\;s^{m}+b_{3}\;s^{3m/2}+b_{4}\;s^{2m}+b_{5}\;{\left(s^{m/2}\right)}_{xx}+b_{6}\;{\left(s^{m}\right)}_{xx},$$

where $b_{j}\neq 0$(1≤j≤6) are real constants.

Form-VI

(f) $$F_{6}\left(s\right)=b_{1}\;s^{m/2}+b_{2}\;s^{m}+b_{3}\;s^{3m/2}+b_{4}\;s^{2m}+b_{5}\;s^{5m/2}+b_{6}\;s^{3m}+b_{7}\;{\left(s^{m/2}\right)}_{xx}+b_{8}\;{\left(s^{m}\right)}_{xx},$$

where $b_{j}\neq 0$(1≤j≤8) are real constants.

In the main text, whenever a specific Kudryashov form is invoked, the corresponding $F_{k}\left(s\right)$ above is used (with $s=|\Phi {|}^{2}$) so that all PDE and reduced ODE coefficients follow uniquely from Eqs. (a)–(f).

***Form-I***

This particular type of refractive index is expressed as

(6) $$F\left(s\right)=\frac{b_{1}}{s^{m}}+\frac{b_{2}}{s^{\frac{m}{2}}}+b_{3}s^{\frac{m}{2}}+b_{4}s^{m},$$

where $b_{j}$ for 1≤j≤4 are non–zero real–valued constants. Consequently, the fundamental model with nonlinear CD is

$$i\Phi_{t}+a{\left({\left|\Phi \right|}^{n}\Phi \right)}_{xx}+\left(\frac{b_{1}}{{\left|\Phi \right|}^{2m}}+\frac{b_{2}}{{\left|\Phi \right|}^{m}}+b_{3}{\left|\Phi \right|}^{m}+b_{4}{\left|\Phi \right|}^{2m}\right)\Phi$$

(7) $$=\alpha \frac{{\left|\Phi_{x}\right|}^{2}}{\Phi^{*}}+\frac{\beta }{4{\left|\Phi \right|}^{2}\Phi^{*}}\left[2{\left|\Phi \right|}^{2}{\left({\left|\Phi \right|}^{2}\right)}_{xx}-{\left\{{\left({\left|\Phi \right|}^{2}\right)}_{x}\right\}}^{2}\right]+\gamma \Phi +\sigma_{1}{\left({\left|\Phi \right|}^{2m}\Phi \right)}_{xx}+\sigma_{2}{\left|\Phi \right|}^{2m}\Phi_{xx}+\sigma_{3}{\left|\Phi \right|}^{2m}\Phi_{xx}^{*}+i\left[\lambda {\left({\left|\Phi \right|}^{2m}\Phi \right)}_{x}+\theta_{1}{\left({\left|\Phi \right|}^{2m}\right)}_{x}\Phi +\theta_{2}{\left|\Phi \right|}^{2m}\Phi_{x}\right].$$

Implementing Eq. (7) as described in (4) simplifies to

$$i\Phi_{t}+a{\left({\left|\Phi \right|}^{n}\Phi \right)}_{xx}+\left(\frac{b_{1}}{{\left|\Phi \right|}^{2m}}+\frac{b_{2}}{{\left|\Phi \right|}^{m}}+b_{3}{\left|\Phi \right|}^{m}+b_{4}{\left|\Phi \right|}^{2m}\right)\Phi$$

$$=\alpha \frac{{\left|\Phi_{x}\right|}^{2}}{\Phi^{*}}+\frac{\beta }{4{\left|\Phi \right|}^{2}\Phi^{*}}\left[2{\left|\Phi \right|}^{2}{\left({\left|\Phi \right|}^{2}\right)}_{xx}-{\left\{{\left({\left|\Phi \right|}^{2}\right)}_{x}\right\}}^{2}\right]+\gamma \Phi +\sigma_{1}{\left({\left|\Phi \right|}^{2m}\Phi \right)}_{xx}+$$

(8) $$\sigma_{2}{\left|\Phi \right|}^{2m}\Phi_{xx}+\sigma_{3}{\left|\Phi \right|}^{2m}\Phi_{xx}^{*}++i\left[\begin{array}{c} \lambda {\left({\left|\Phi \right|}^{2m}\Phi \right)}_{x}+\theta_{1}{\left({\left|\Phi \right|}^{2m}\right)}_{x}\Phi -\\ \left\{2m\theta_{1}+\left(2m+1\right)\lambda \right\}{\left|\Phi \right|}^{2m}\Phi_{x} \end{array}\right].$$

Substituting (2) into (7), Eq. (3) reduces to

$$a\left(n+1\right)g^{n}\left(x\right)\left[g\left(x\right)g^{\prime \prime }\left(x\right)+n{\left\{g^{\prime }\left(x\right)\right\}}^{2}\right]-\left[\gamma +\omega -\frac{b_{1}}{g^{2m}\left(x\right)}-\frac{b_{2}}{g^{m}\left(x\right)}-b_{3}g^{m}\left(x\right)-b_{4}g^{2m}\left(x\right)\right]g^{2}\left(x\right)$$

$$-\left\{\left(2m+1\right)\sigma_{1}+\sigma_{2}-\sigma_{3}\right\}g^{2m+1}\left(x\right)g^{\prime \prime }\left(x\right)-2m\left(2m+1\right)\sigma_{1}g^{2m}\left(x\right){\left\{g^{\prime }\left(x\right)\right\}}^{2}$$

(9) $$-\alpha {\left\{g^{\prime }\left(x\right)\right\}}^{2}-\beta g\left(x\right)g^{\prime \prime }\left(x\right)=0.$$

Eq. (9) demonstrates a translational Lie symmetry represented by ∂/∂x. When this symmetry is applied, it allows for the integration of (9), resulting in an implicit nonlocal solution

(10) $$x=\int \;\sqrt{\frac{A}{B}}\;dg,$$

where

(11) $$A=\text{exp}\left(-2\int \;-\frac{2m\left(2m+1\right)\sigma_{1}\tau_{1}^{2m}-an\left(n+1\right)\tau_{1}^{n}+\alpha }{\tau_{1}\left(\left(\left(2m+1\right)\sigma_{1}+\sigma_{2}+\sigma_{3}\right)\tau_{1}^{2m}-a\left(n+1\right)\tau_{1}^{n}+\beta \right)}d\tau_{1}\right),$$

and

(12) $$B=2\int \;\frac{\Gamma \tau_{2}\left(b_{1}\tau_{2}^{-2m}+b_{2}\tau_{2}^{-m}+\left(b_{4}\tau_{2}^{m}+b_{3}\right)\tau_{2}^{m}-\gamma -\omega \right)}{\left(\left(2m+1\right)\sigma_{1}+\sigma_{2}+\sigma_{3}\right)\tau_{2}^{2m}-a\left(n+1\right)\tau_{2}^{n}+\beta }d\tau_{2},$$

and

(13) $$\Gamma =\text{exp}\left(-2\int \;-\frac{2m\left(2m+1\right)\sigma_{1}\tau_{1}^{2m}-an\left(n+1\right)\tau_{1}^{n}+\alpha }{\tau_{1}\left(\left(\left(2m+1\right)\sigma_{1}+\sigma_{2}+\sigma_{3}\right)\tau_{1}^{2m}-a\left(n+1\right)\tau_{1}^{n}+\beta \right)}d\tau_{1}\right).$$

The solution structure (10) poses a parameter constraint for the existence of the solution, namely

(14) AB>0.

Eq. (9) is autonomous in the traveling variable (it contains no explicit ξ), which reflects the translational Lie symmetry ∂/∂ξ. Consequently, one may reduce the order by setting

$p\left(g\right)=\frac{dg}{d\xi }=g{}^{\prime }\left(\xi \right),\;g{}^{\prime \prime }\left(\xi \right)=\frac{dp}{d\xi }=p\left(g\right)\frac{dp}{dg}.$ (a)

Substituting (a) into (9) yields a first–order equation in p(g). It is convenient to introduce $Y\left(g\right)=p{\left(g\right)}^{2}$, so that

$\frac{dY}{dg}=2p\frac{dp}{dg}.$ (b)

After simplification, one obtains a linear first–order ODE of the form

$\frac{dY}{dg}+P\left(g\right)\;Y=Q\left(g\right),$ (c) where P(g) and Q(g) are explicit rational functions of g formed from the coefficients in (9). The integrating factor is

$\mu \left(g\right)=\text{exp}\left(\int_{0}^{g}P\left(\tau_{1}\right)\;d\tau_{1}\right).$ (d)

Thus,

$Y\left(g\right)=\mu {\left(g\right)}^{-1}\left(C+\int_{0}^{g}\mu \left(\tau_{2}\right)\;Q\left(\tau_{2}\right)\;d\tau_{2}\right),$ (e) and since $Y\left(g\right)={\left(\frac{dg}{d\xi }\right)}^{2}$, we obtain the quadrature

$\xi -\xi_{0}=\int_{0}^{g}\sqrt{\frac{\mu (s)}{\;C+\int_{0}^{g}\mu \left(\tau_{2}\right)\;Q\left(\tau_{2}\right)\;d\tau_{2}\;}}\;ds,$ (f) where C and $\xi_{0}$ are independent constants of integration. Comparing (f) with Eq. (10), we may identify

$A\left(g\right)=\mu \left(g\right),\;B\left(g\right)=C+\int_{0}^{g}\mu \left(\tau_{2}\right)\;Q\left(\tau_{2}\right)\;d\tau_{2},$ (g) which yields Eq. (10).

***Form-II***

The law of refractive index for the second form is defined as follows:

(15) $$F\left(s\right)=\frac{b_{1}}{s^{2m}}+\frac{b_{2}}{s^{\frac{3m}{2}}}+\frac{b_{3}}{s^{m}}+\frac{b_{4}}{s^{\frac{m}{2}}}+b_{5}s^{\frac{m}{2}}+b_{6}s^{m}+b_{7}s^{\frac{3m}{2}}+b_{8}s^{2m},$$

for nonzero real-valued constants $b_{j}$ where 1≤j≤8. Hence, the governing perturbed complex Ginzburg-Landau equation with nonlinear CD is

$$i\Phi_{t}+a{\left({\left|\Phi \right|}^{n}\Phi \right)}_{xx}+\left(\frac{b_{1}}{{\left|\Phi \right|}^{4m}}+\frac{b_{2}}{{\left|\Phi \right|}^{3m}}+\frac{b_{3}}{{\left|\Phi \right|}^{2m}}+\frac{b_{4}}{{\left|\Phi \right|}^{m}}+b_{5}{\left|\Phi \right|}^{m}+b_{6}{\left|\Phi \right|}^{2m}+b_{7}{\left|\Phi \right|}^{3m}+b_{8}{\left|\Phi \right|}^{4m}\right)\Phi$$

(16) $$=\alpha \frac{{\left|\Phi_{x}\right|}^{2}}{\Phi^{*}}+\frac{\beta }{4{\left|\Phi \right|}^{2}\Phi^{*}}\left[2{\left|\Phi \right|}^{2}{\left({\left|\Phi \right|}^{2}\right)}_{xx}-{\left\{{\left({\left|\Phi \right|}^{2}\right)}_{x}\right\}}^{2}\right]+\gamma \Phi +\sigma_{1}{\left({\left|\Phi \right|}^{2m}\Phi \right)}_{xx}+\sigma_{2}{\left|\Phi \right|}^{2m}\Phi_{xx}+\sigma_{3}{\left|\Phi \right|}^{2m}\Phi_{xx}^{*}+i\left[\lambda {\left({\left|\Phi \right|}^{2m}\Phi \right)}_{x}+\theta_{1}{\left({\left|\Phi \right|}^{2m}\right)}_{x}\Phi +\theta_{2}{\left|\Phi \right|}^{2m}\Phi_{x}\right]$$

Next, implementing (4), Eq. (16) simplifies to

$$i\Phi_{t}+a{\left({\left|\Phi \right|}^{n}\Phi \right)}_{xx}+\left(\frac{b_{1}}{{\left|\Phi \right|}^{4m}}+\frac{b_{2}}{{\left|\Phi \right|}^{3m}}+\frac{b_{3}}{{\left|\Phi \right|}^{2m}}+\frac{b_{4}}{{\left|\Phi \right|}^{m}}+b_{5}{\left|\Phi \right|}^{m}+b_{6}{\left|\Phi \right|}^{2m}+b_{7}{\left|\Phi \right|}^{3m}+\;b_{8}{\left|\Phi \right|}^{4m}\right)\Phi$$

$$=\alpha \frac{{\left|\Phi_{x}\right|}^{2}}{\Phi^{*}}+\frac{\beta }{4{\left|\Phi \right|}^{2}\Phi^{*}}\left[2{\left|\Phi \right|}^{2}{\left({\left|\Phi \right|}^{2}\right)}_{xx}-{\left\{{\left({\left|\Phi \right|}^{2}\right)}_{x}\right\}}^{2}\right]+\gamma \Phi +\sigma_{1}{\left({\left|\Phi \right|}^{2m}\Phi \right)}_{xx}+$$

(17) $$\sigma_{2}{\left|\Phi \right|}^{2m}\Phi_{xx}+\sigma_{3}{\left|\Phi \right|}^{2m}\Phi_{xx}^{*}+i\left[\begin{array}{c} \lambda {\left({\left|\Phi \right|}^{2m}\Phi \right)}_{x}+\theta_{1}{\left({\left|\Phi \right|}^{2m}\right)}_{x}\Phi \\ -\left\{2m\theta_{1}+\left(2m+1\right)\lambda \right\}{\left|\Phi \right|}^{2m}\Phi_{x} \end{array}\right].$$

Substituting into (17), Eq. (9) reads:

$$a\left(n+1\right)g^{n}\left(x\right)\left[g\left(x\right)g^{\prime \prime }\left(x\right)+n{\left\{g^{\prime }\left(x\right)\right\}}^{2}\right]$$

$$-\left[\gamma +\omega -\frac{b_{1}}{g^{4m}\left(x\right)}-\frac{b_{2}}{g^{3m}\left(x\right)}-\frac{b_{3}}{g^{2m}\left(x\right)}-\frac{b_{4}}{g^{m}\left(x\right)}-b_{5}g^{m}\left(x\right)-b_{6}g^{2m}\left(x\right)-b_{7}g^{3m}\left(x\right)-b_{8}g^{4m}\left(x\right)\right]g^{2}\left(x\right)$$

$$-\left\{\left(2m+1\right)\sigma_{1}+\sigma_{2}-\sigma_{3}\right\}g^{2m+1}\left(x\right)g^{\prime \prime }\left(x\right)-2m\left(2m+1\right)\sigma_{1}g^{2m}\left(x\right){\left\{g^{\prime }\left(x\right)\right\}}^{2}$$

(18) $$-\alpha {\left\{g^{\prime }\left(x\right)\right\}}^{2}-\beta g\left(x\right)g^{\prime \prime }\left(x\right)=0.$$

Eq. (18) has the same translational Lie symmetry as the first formulation of SPM. The application of this symmetry results in the implicit solution (10), where

(19) $$A=\text{exp}\left(-2\int \;-\frac{2m\left(2m+1\right)\sigma_{1}\tau_{1}^{2m}-an\left(n+1\right)\tau_{1}^{n}+\alpha }{\tau_{1}\left(\left(\left(2m+1\right)\sigma_{1}+\sigma_{2}+\sigma_{3}\right)\tau_{1}^{2m}-a\left(n+1\right)\tau_{1}^{n}+\beta \right)}d\tau_{1}\right),$$

(20) $$B=2\int \;\frac{\Gamma \tau_{2}^{1-4m}\left(\left(\left(\left(-\left(\left(\gamma +\omega \right)\tau_{2}^{m}\right)+\left(\left(\left(b_{8}\tau_{2}^{m}+b_{7}\right)\tau_{2}^{m}+b_{6}\right)\tau_{2}^{m}+b_{5}\right)\tau_{2}^{2m}+b_{4}\right)\tau_{2}^{m}+b_{3}\right)\tau_{2}^{m}+b_{2}\right)\tau_{2}^{m}+b_{1}\right)}{\left(\left(2m+1\right)\sigma_{1}+\sigma_{2}+\sigma_{3}\right)\tau_{2}^{2m}-a\left(n+1\right)\tau_{2}^{n}+\beta }d\tau_{2},$$

and

(21) $$\Gamma =\text{exp}\left(-2\int \;-\frac{2m\left(2m+1\right)\sigma_{1}\tau_{1}^{2m}-an\left(n+1\right)\tau_{1}^{n}+\alpha }{\tau_{1}\left(\left(\left(2m+1\right)\sigma_{1}+\sigma_{2}+\sigma_{3}\right)\tau_{1}^{2m}-a\left(n+1\right)\tau_{1}^{n}+\beta \right)}d\tau_{1}\right).$$

The solution (10) will exist provided (14) holds where in this case A and B are given by (19) and (20) respectively.

***Form-III***

The principle of refractive index is as follows:

(22) $$F\left(s\right)=b_{1}s^{\frac{m}{2}}+b_{2}s^{m}+b_{3}{\left(s^{\frac{m}{2}}\right)}^{\prime \prime }.$$

Consequently, governing laws perturbed CGLE assumes the following form

$$i\Phi_{t}+a{\left({\left|\Phi \right|}^{n}\Phi \right)}_{xx}+\left[b_{1}{\left|\Phi \right|}^{m}+b_{2}{\left|\Phi \right|}^{2m}+b_{3}{\left({\left|\Phi \right|}^{m}\right)}_{xx}\right]\Phi$$

$$=\alpha \frac{{\left|\Phi_{x}\right|}^{2}}{\Phi^{*}}+\frac{\beta }{4{\left|\Phi \right|}^{2}\Phi^{*}}\left[2{\left|\Phi \right|}^{2}{\left({\left|\Phi \right|}^{2}\right)}_{xx}-{\left\{{\left({\left|\Phi \right|}^{2}\right)}_{x}\right\}}^{2}\right]+\gamma \Phi +\sigma_{1}{\left({\left|\Phi \right|}^{2m}\Phi \right)}_{xx}+\sigma_{2}{\left|\Phi \right|}^{2m}\Phi_{xx}+\sigma_{3}{\left|\Phi \right|}^{2m}\Phi_{xx}^{*}+$$

(23) $$+i\left[\lambda {\left({\left|\Phi \right|}^{2m}\Phi \right)}_{x}+\theta_{1}{\left({\left|\Phi \right|}^{2m}\right)}_{x}\Phi +\theta_{2}{\left|\Phi \right|}^{2m}\Phi_{x}\right]$$

which, after the application of (4), assumes the form

$$i\Phi_{t}+a{\left({\left|\Phi \right|}^{n}\Phi \right)}_{xx}+\left[b_{1}{\left|\Phi \right|}^{m}+b_{2}{\left|\Phi \right|}^{2m}+b_{3}{\left({\left|\Phi \right|}^{m}\right)}_{xx}\right]\Phi$$

(24) $$=\alpha \frac{{\left|\Phi_{x}\right|}^{2}}{\Phi^{*}}+\frac{\beta }{4{\left|\Phi \right|}^{2}\Phi^{*}}\left[2{\left|\Phi \right|}^{2}{\left({\left|\Phi \right|}^{2}\right)}_{xx}-{\left\{{\left({\left|\Phi \right|}^{2}\right)}_{x}\right\}}^{2}\right]+\gamma \Phi +\sigma_{1}{\left({\left|\Phi \right|}^{2m}\Phi \right)}_{xx}+\sigma_{2}{\left|\Phi \right|}^{2m}\Phi_{xx}+\sigma_{3}{\left|\Phi \right|}^{2m}\Phi_{xx}^{*}+i\left[\lambda {\left({\left|\Phi \right|}^{2m}\Phi \right)}_{x}+\theta_{1}{\left({\left|\Phi \right|}^{2m}\right)}_{x}\Phi -\left\{2m\theta_{1}+\left(2m+1\right)\lambda \right\}{\left|\Phi \right|}^{2m}\Phi_{x}\right].$$

By substituting (2) into (24) and simplifying the real part, we obtain

$$a\left(n+1\right)g^{n}\left(x\right)\left[g\left(x\right)g^{\prime \prime }\left(x\right)+n{\left\{g^{\prime }\left(x\right)\right\}}^{2}\right]-\left[\gamma +\omega -b_{1}g^{m}\left(x\right)-b_{2}g^{2m}\left(x\right)-b_{3}{\left\{g^{m}\left(x\right)\right\}}^{\prime \prime }\right]g^{2}\left(x\right)$$

$$-\left\{\left(2m+1\right)\sigma_{1}+\sigma_{2}-\sigma_{3}\right\}g^{2m+1}\left(x\right)g^{\prime \prime }\left(x\right)-2m\left(2m+1\right)\sigma_{1}g^{2m}\left(x\right){\left\{g^{\prime }\left(x\right)\right\}}^{2}$$

(25) $$-\alpha {\left\{g^{\prime }\left(x\right)\right\}}^{2}-\beta g\left(x\right)g^{\prime \prime }\left(x\right)=0.$$

The application of the translational Lie symmetry to (26) results in the implicit solution as presented in (10), where in the present case

(26) $$A=\text{exp}\left(-2\int \;\frac{m\left(2\left(2m+1\right)\sigma_{1}\tau_{1}^{m}-\left(m-1\right)b_{3}\right)\tau_{1}^{m}-an\left(n+1\right)\tau_{1}^{n}+\alpha }{\tau_{1}\left(mb_{3}\tau_{1}^{m}-\left(\left(2m+1\right)\sigma_{1}+\sigma_{2}+\sigma_{3}\right)\tau_{1}^{2m}+a\left(n+1\right)\tau_{1}^{n}-\beta \right)}d\tau_{1}\right),$$

and

(27) $$B=2\int \;-\frac{\text{exp}\left(-2\int \;\frac{m\left(2\left(2m+1\right)\sigma_{1}\tau_{1}^{m}-\left(m-1\right)b_{3}\right)\tau_{1}^{m}-an\left(n+1\right)\tau_{1}^{n}+\alpha }{\tau_{1}\left(mb_{3}\tau_{1}^{m}-\left(\left(2m+1\right)\sigma_{1}+\sigma_{2}+\sigma_{3}\right)\tau_{1}^{2m}+a\left(n+1\right)\tau_{1}^{n}-\beta \right)}d\tau_{1}\right)\tau_{2}\left(-\left(\left(b_{2}\tau_{2}^{m}+b_{1}\right)\tau_{2}^{m}\right)+\gamma +\omega \right)}{-mb_{3}\tau_{2}^{m}+\left(\left(2m+1\right)\sigma_{1}+\sigma_{2}+\sigma_{3}\right)\tau_{2}^{2m}-a\left(n+1\right)\tau_{2}^{n}+\beta }d\tau_{2}.$$

The condition specified in (14) must be satisfied for the implicit solution presented in (10) to be acceptable.

***Form-IV***

The refractive index law applicable to this form is as follows

(28) $$F\left(s\right)=b_{1}s^{\frac{m}{2}}+b_{2}s^{m}+b_{3}s^{\frac{3m}{2}}+b_{4}s^{2m}+b_{5}s^{\frac{5m}{2}}+b_{6}s^{3m},$$

for real-valued constants $b_{j}$ where 1≤j≤6. Consequently, the governing model is expressed as

$$i\Phi_{t}+a{\left({\left|\Phi \right|}^{n}\Phi \right)}_{xx}+\left(b_{1}{\left|\Phi \right|}^{m}+b_{2}{\left|\Phi \right|}^{2m}+b_{3}{\left|\Phi \right|}^{3m}+b_{4}{\left|\Phi \right|}^{4m}+b_{5}{\left|\Phi \right|}^{5m}+b_{6}{\left|\Phi \right|}^{6m}\right)\Phi$$

$$=\alpha \frac{{\left|\Phi_{x}\right|}^{2}}{\Phi^{*}}+\frac{\beta }{4{\left|\Phi \right|}^{2}\Phi^{*}}\left[2{\left|\Phi \right|}^{2}{\left({\left|\Phi \right|}^{2}\right)}_{xx}-{\left\{{\left({\left|\Phi \right|}^{2}\right)}_{x}\right\}}^{2}\right]+\gamma \Phi +\sigma_{1}{\left({\left|\Phi \right|}^{2m}\Phi \right)}_{xx}+\sigma_{2}{\left|\Phi \right|}^{2m}\Phi_{xx}+\sigma_{3}{\left|\Phi \right|}^{2m}\Phi_{xx}^{*}$$

(29) $$+i\left[\lambda {\left({\left|\Phi \right|}^{2m}\Phi \right)}_{x}+\theta_{1}{\left({\left|\Phi \right|}^{2m}\right)}_{x}\Phi +\theta_{2}{\left|\Phi \right|}^{2m}\Phi_{x}\right],$$

which by virtue of (4) takes the form

$$i\Phi_{t}+a{\left({\left|\Phi \right|}^{n}\Phi \right)}_{xx}+\left(b_{1}{\left|\Phi \right|}^{m}+b_{2}{\left|\Phi \right|}^{2m}+b_{3}{\left|\Phi \right|}^{3m}+b_{4}{\left|\Phi \right|}^{4m}+b_{5}{\left|\Phi \right|}^{5m}+b_{6}{\left|\Phi \right|}^{6m}\right)\Phi$$

(30) $$=\alpha \frac{{\left|\Phi_{x}\right|}^{2}}{\Phi^{*}}+\frac{\beta }{4{\left|\Phi \right|}^{2}\Phi^{*}}\left[2{\left|\Phi \right|}^{2}{\left({\left|\Phi \right|}^{2}\right)}_{xx}-{\left\{{\left({\left|\Phi \right|}^{2}\right)}_{x}\right\}}^{2}\right]+\gamma \Phi +\sigma_{1}{\left({\left|\Phi \right|}^{2m}\Phi \right)}_{xx}+\sigma_{2}{\left|\Phi \right|}^{2m}\Phi_{xx}+\sigma_{3}{\left|\Phi \right|}^{2m}\Phi_{xx}^{*}+i\left[\lambda {\left({\left|\Phi \right|}^{2m}\Phi \right)}_{x}+\theta_{1}{\left({\left|\Phi \right|}^{2m}\right)}_{x}\Phi -\left\{2m\theta_{1}+\left(2m+1\right)\lambda \right\}{\left|\Phi \right|}^{2m}\Phi_{x}\right].$$

Inserting (2) into (30) yields the real component as follows:

$$a\left(n+1\right)g^{n}\left(x\right)\left[g\left(x\right)g^{\prime \prime }\left(x\right)+n{\left\{g^{\prime }\left(x\right)\right\}}^{2}\right]$$

$$-\left[\gamma +\omega -\frac{b_{1}}{g^{m}\left(x\right)}-b_{2}g^{2m}\left(x\right)-b_{3}g^{3m}\left(x\right)-b_{4}g^{4m}\left(x\right)-b_{5}g^{5m}\left(x\right)-b_{6}g^{6m}\left(x\right)\right]g^{2}\left(x\right)$$

$$-\left\{\left(2m+1\right)\sigma_{1}+\sigma_{2}-\sigma_{3}\right\}g^{2m+1}\left(x\right)g^{\prime \prime }\left(x\right)-2m\left(2m+1\right)\sigma_{1}g^{2m}\left(x\right){\left\{g^{\prime }\left(x\right)\right\}}^{2}$$

(31) $$-\alpha {\left\{g^{\prime }\left(x\right)\right\}}^{2}-\beta g\left(x\right)g^{\prime \prime }\left(x\right)=0.$$

Implementing the translational Lie symmetry, Eq. (31) integrates to (10) along with the parametric constraint (14) where

(32) $$A=\text{exp}\left(-2\int \;-\frac{2m\left(2m+1\right)\sigma_{1}\tau_{1}^{2m}-an\left(n+1\right)\tau_{1}^{n}+\alpha }{\tau_{1}\left(\left(\left(2m+1\right)\sigma_{1}+\sigma_{2}+\sigma_{3}\right)\tau_{1}^{2m}-a\left(n+1\right)\tau_{1}^{n}+\beta \right)}d\tau_{1}\right),$$

(33) $$B=2\int \;-\frac{\text{exp}\left(-2\int \;-\frac{2m\left(2m+1\right)\sigma_{1}\tau_{1}^{2m}-an\left(n+1\right)\tau_{1}^{n}+\alpha }{\tau_{1}\left(\left(\left(2m+1\right)\sigma_{1}+\sigma_{2}+\sigma_{3}\right)\tau_{1}^{2m}-a\left(n+1\right)\tau_{1}^{n}+\beta \right)}d\tau_{1}\right)\Gamma }{\left(\left(2m+1\right)\sigma_{1}+\sigma_{2}+\sigma_{3}\right)\tau_{2}^{2m}-a\left(n+1\right)\tau_{2}^{n}+\beta }d\tau_{2},$$

and

(34) $$\Gamma =\tau_{2}\left(-\left(\tau_{2}^{m}\left(\tau_{2}^{m}\left(\tau_{2}^{m}\left(\tau_{2}^{m}\left(\tau_{2}^{m}\left(b_{6}\tau_{2}^{m}+b_{5}\right)+b_{4}\right)+b_{3}\right)+b_{2}\right)+b_{1}\right)\right)+\gamma +\omega \right).$$

***Form-V***

In this scenario, the law of the refractive index is articulated as:

(35) $$F\left(s\right)=b_{1}s^{\frac{m}{2}}+b_{2}s^{m}+b_{3}s^{\frac{3m}{2}}+b_{4}s^{2m}+b_{5}{\left(s^{\frac{m}{2}}\right)}^{\prime \prime }+b_{6}{\left(s^{m}\right)}^{\prime \prime }.$$

The equation that governs CGLE is thus expressed as

$$i\Phi_{t}+a{\left({\left|\Phi \right|}^{n}\Phi \right)}_{xx}+\left[b_{1}{\left|\Phi \right|}^{m}+b_{2}{\left|\Phi \right|}^{2m}+b_{3}{\left|\Phi \right|}^{3m}+b_{4}{\left|\Phi \right|}^{4m}+b_{5}{\left({\left|\Phi \right|}^{m}\right)}_{xx}+b_{6}{\left({\left|\Phi \right|}^{2m}\right)}_{xx}\right]\Phi$$

$$=\alpha \frac{{\left|\Phi_{x}\right|}^{2}}{\Phi^{*}}+\frac{\beta }{4{\left|\Phi \right|}^{2}\Phi^{*}}\left[2{\left|\Phi \right|}^{2}{\left({\left|\Phi \right|}^{2}\right)}_{xx}-{\left\{{\left({\left|\Phi \right|}^{2}\right)}_{x}\right\}}^{2}\right]+\gamma \Phi +\sigma_{1}{\left({\left|\Phi \right|}^{2m}\Phi \right)}_{xx}+\sigma_{2}{\left|\Phi \right|}^{2m}\Phi_{xx}+\sigma_{3}{\left|\Phi \right|}^{2m}\Phi_{xx}^{*}$$

(36) $$+i\left[\lambda {\left({\left|\Phi \right|}^{2m}\Phi \right)}_{x}+\theta_{1}{\left({\left|\Phi \right|}^{2m}\right)}_{x}\Phi +\theta_{2}{\left|\Phi \right|}^{2m}\Phi_{x}\right]$$

which reduces to

$$i\Phi_{t}+a{\left({\left|\Phi \right|}^{n}\Phi \right)}_{xx}+\left[b_{1}{\left|\Phi \right|}^{m}+b_{2}{\left|\Phi \right|}^{2m}+b_{3}{\left|\Phi \right|}^{3m}+b_{4}{\left|\Phi \right|}^{4m}+b_{5}{\left({\left|\Phi \right|}^{m}\right)}_{xx}+b_{6}{\left({\left|\Phi \right|}^{2m}\right)}_{xx}\right]\Phi$$

$$=\alpha \frac{{\left|\Phi_{x}\right|}^{2}}{\Phi^{*}}+\frac{\beta }{4{\left|\Phi \right|}^{2}\Phi^{*}}\left[2{\left|\Phi \right|}^{2}{\left({\left|\Phi \right|}^{2}\right)}_{xx}-{\left\{{\left({\left|\Phi \right|}^{2}\right)}_{x}\right\}}^{2}\right]+\gamma \Phi +\sigma_{1}{\left({\left|\Phi \right|}^{2m}\Phi \right)}_{xx}+\sigma_{2}{\left|\Phi \right|}^{2m}\Phi_{xx}+\sigma_{3}{\left|\Phi \right|}^{2m}\Phi_{xx}^{*}$$

(37) $$+i\left[\lambda {\left({\left|\Phi \right|}^{2m}\Phi \right)}_{x}+\theta_{1}{\left({\left|\Phi \right|}^{2m}\right)}_{x}\Phi -\left\{2m\theta_{1}+\left(2m+1\right)\lambda \right\}{\left|\Phi \right|}^{2m}\Phi_{x}\right],$$

after making use of (4). Next, substituting (2) into (37), the real part leaves us with

$$a\left(n+1\right)g^{n}\left(x\right)\left[g\left(x\right)g^{\prime \prime }\left(x\right)+n{\left\{g^{\prime }\left(x\right)\right\}}^{2}\right]$$

$$-\left[\gamma +\omega -\frac{b_{1}}{g^{m}\left(x\right)}-b_{2}g^{2m}\left(x\right)-b_{3}g^{3m}\left(x\right)-b_{4}g^{4m}\left(x\right)-b_{5}{\left\{g^{m}\left(x\right)\right\}}^{\prime \prime }-b_{6}{\left\{g^{2m}\left(x\right)\right\}}^{\prime \prime }\right]g^{2}\left(x\right)$$

$$-\left\{\left(2m+1\right)\sigma_{1}+\sigma_{2}-\sigma_{3}\right\}g^{2m+1}\left(x\right)g^{\prime \prime }\left(x\right)-2m\left(2m+1\right)\sigma_{1}g^{2m}\left(x\right){\left\{g^{\prime }\left(x\right)\right\}}^{2}$$

(38) $$-\alpha {\left\{g^{\prime }\left(x\right)\right\}}^{2}-\beta g\left(x\right)g^{\prime \prime }\left(x\right)=0.$$

The translational Lie symmetry integrates (38) to (10) with the constraint condition (14) where for this form of SPM

(39) $$A=\text{exp}\left(-2\int \;-\frac{m\left(-2\left(\left(2m-1\right)b_{6}-\left(2m+1\right)\sigma_{1}\right)\tau_{1}^{m}-\left(m-1\right)b_{5}\right)\tau_{1}^{m}-an\left(n+1\right)\tau_{1}^{n}+\alpha }{\tau_{1}\left(-mb_{5}\tau_{1}^{m}+\left(-2mb_{6}+2m\sigma_{1}+\sigma_{1}+\sigma_{2}+\sigma_{3}\right)\tau_{1}^{2m}-a\left(n+1\right)\tau_{1}^{n}+\beta \right)}d\tau_{1}\right),$$

(40) $$B=2\int \;\frac{\text{exp}\left(-2\int \;-\frac{m\left(-2\left(\left(2m-1\right)b_{6}-\left(2m+1\right)\sigma_{1}\right)\tau_{1}^{m}-\left(m-1\right)b_{5}\right)\tau_{1}^{m}-an\left(n+1\right)\tau_{1}^{n}+\alpha }{\tau_{1}\left(-mb_{5}\tau_{1}^{m}+\left(-2mb_{6}+2m\sigma_{1}+\sigma_{1}+\sigma_{2}+\sigma_{3}\right)\tau_{1}^{2m}-a\left(n+1\right)\tau_{1}^{n}+\beta \right)}d\tau_{1}\right)\Gamma }{mb_{5}\tau_{2}^{m}-\left(-2mb_{6}+\left(2m+1\right)\sigma_{1}+\sigma_{2}+\sigma_{3}\right)\tau_{2}^{2m}+a\left(n+1\right)\tau_{2}^{n}-\beta }d\tau_{2},$$

with

(41) $$\Gamma =\tau_{2}\left(-\left(\tau_{2}^{m}\left(\tau_{2}^{m}\left(\tau_{2}^{m}\left(b_{4}\tau_{2}^{m}+b_{3}\right)+b_{2}\right)+b_{1}\right)\right)+\gamma +\omega \right).$$

***Form-VI***

The law of refractive index is formulated as follows:

(42) $$F\left(s\right)=b_{1}s^{\frac{m}{2}}+b_{2}s^{m}+b_{3}s^{\frac{3m}{2}}+b_{4}s^{2m}+b_{5}s^{\frac{5m}{2}}+b_{6}s^{3m}+b_{7}{\left(s^{\frac{m}{2}}\right)}^{\prime \prime }+b_{8}{\left(s^{m}\right)}^{\prime \prime }.$$

In this context, $b_{j}$ represents real-valued constants for the range 1≤j≤8. This results in the governing CGLE being expressed as:

$$i\Phi_{t}+a{\left({\left|\Phi \right|}^{n}\Phi \right)}_{xx}+\left[b_{1}{\left|\Phi \right|}^{m}+b_{2}{\left|\Phi \right|}^{2m}+b_{3}{\left|\Phi \right|}^{3m}+b_{4}{\left|\Phi \right|}^{4m}+b_{5}{\left|\Phi \right|}^{5m}+b_{6}{\left|\Phi \right|}^{6m}+b_{7}{\left({\left|\Phi \right|}^{m}\right)}_{xx}+b_{8}{\left({\left|\Phi \right|}^{2m}\right)}_{xx}\right]\Phi$$

(43) $$=\alpha \frac{{\left|\Phi_{x}\right|}^{2}}{\Phi^{*}}+\frac{\beta }{4{\left|\Phi \right|}^{2}\Phi^{*}}\left[2{\left|\Phi \right|}^{2}{\left({\left|\Phi \right|}^{2}\right)}_{xx}-{\left\{{\left({\left|\Phi \right|}^{2}\right)}_{x}\right\}}^{2}\right]+\gamma \Phi +\sigma_{1}{\left({\left|\Phi \right|}^{2m}\Phi \right)}_{xx}+\sigma_{2}{\left|\Phi \right|}^{2m}\Phi_{xx}+\sigma_{3}{\left|\Phi \right|}^{2m}\Phi_{xx}^{*}+i\left[\lambda {\left({\left|\Phi \right|}^{2m}\Phi \right)}_{x}+\theta_{1}{\left({\left|\Phi \right|}^{2m}\right)}_{x}\Phi +\theta_{2}{\left|\Phi \right|}^{2m}\Phi_{x}\right],$$

which, by virtue of (4), changes to

$$i\Phi_{t}+a{\left({\left|\Phi \right|}^{n}\Phi \right)}_{xx}+\left[b_{1}{\left|\Phi \right|}^{m}+b_{2}{\left|\Phi \right|}^{2m}+b_{3}{\left|\Phi \right|}^{3m}+b_{4}{\left|\Phi \right|}^{4m}+b_{5}{\left|\Phi \right|}^{5m}+b_{6}{\left|\Phi \right|}^{6m}+b_{7}{\left({\left|\Phi \right|}^{m}\right)}_{xx}+b_{8}{\left({\left|\Phi \right|}^{2m}\right)}_{xx}\right]\Phi$$

$$=\alpha \frac{{\left|\Phi_{x}\right|}^{2}}{\Phi^{*}}+\frac{\beta }{4{\left|\Phi \right|}^{2}\Phi^{*}}\left[2{\left|\Phi \right|}^{2}{\left({\left|\Phi \right|}^{2}\right)}_{xx}-{\left\{{\left({\left|\Phi \right|}^{2}\right)}_{x}\right\}}^{2}\right]+\gamma \Phi +\sigma_{1}{\left({\left|\Phi \right|}^{2m}\Phi \right)}_{xx}+\sigma_{2}{\left|\Phi \right|}^{2m}\Phi_{xx}+\sigma_{3}{\left|\Phi \right|}^{2m}\Phi_{xx}^{*}+$$

(44) $$+i\left[\lambda {\left({\left|\Phi \right|}^{2m}\Phi \right)}_{x}+\theta_{1}{\left({\left|\Phi \right|}^{2m}\right)}_{x}\Phi -\left\{2m\theta_{1}+\left(2m+1\right)\lambda \right\}{\left|\Phi \right|}^{2m}\Phi_{x}\right].$$

Likewise, as in the preceding scenarios of SPM, the ODE for g(x) is determined to be

$$a\left(n+1\right)g^{n}\left(x\right)\left[g\left(x\right)g^{\prime \prime }\left(x\right)+n{\left\{g^{\prime }\left(x\right)\right\}}^{2}\right]$$

$$-\left[\gamma +\omega -\frac{b_{1}}{g^{m}\left(x\right)}-b_{2}g^{2m}\left(x\right)-b_{3}g^{3m}\left(x\right)-b_{4}g^{4m}\left(x\right)-b_{5}g^{5m}\left(x\right)-b_{6}g^{6m}\left(x\right)-b_{7}{\left\{g^{m}\left(x\right)\right\}}^{\prime \prime }-b_{8}{\left\{g^{2m}\left(x\right)\right\}}^{\prime \prime }\right]g^{2}\left(x\right)$$

(45) $$-\left\{\left(2m+1\right)\sigma_{1}+\sigma_{2}-\sigma_{3}\right\}g^{2m+1}\left(x\right)g^{\prime \prime }\left(x\right)-2m\left(2m+1\right)\sigma_{1}g^{2m}\left(x\right){\left\{g^{\prime }\left(x\right)\right\}}^{2}-\alpha {\left\{g^{\prime }\left(x\right)\right\}}^{2}-\beta g\left(x\right)g^{\prime \prime }\left(x\right)=0.$$

The translational Lie symmetry integrates this ODE to (10) with the constraints as in (14) where in this case

(46) $$A=\text{exp}\left(-2\int \;-\frac{m\left(-2\left(\left(2m-1\right)b_{8}-\left(2m+1\right)\sigma_{1}\right)\tau_{1}^{m}-\left(m-1\right)b_{7}\right)\tau_{1}^{m}-an\left(n+1\right)\tau_{1}^{n}+\alpha }{\tau_{1}\left(-mb_{7}\tau_{1}^{m}+\left(-2mb_{8}+2m\sigma_{1}+\sigma_{1}+\sigma_{2}+\sigma_{3}\right)\tau_{1}^{2m}-a\left(n+1\right)\tau_{1}^{n}+\beta \right)}d\tau_{1}\right),$$

(47) $$B=2\int \;\frac{\text{exp}\left(-2\int \;-\frac{m\left(-2\left(\left(2m-1\right)b_{8}-\left(2m+1\right)\sigma_{1}\right)\tau_{1}^{m}-\left(m-1\right)b_{7}\right)\tau_{1}^{m}-an\left(n+1\right)\tau_{1}^{n}+\alpha }{\tau_{1}\left(-mb_{7}\tau_{1}^{m}+\left(-2mb_{8}+2m\sigma_{1}+\sigma_{1}+\sigma_{2}+\sigma_{3}\right)\tau_{1}^{2m}-a\left(n+1\right)\tau_{1}^{n}+\beta \right)}d\tau_{1}\right)\Gamma }{mb_{7}\tau_{2}^{m}-\left(-2mb_{8}+\left(2m+1\right)\sigma_{1}+\sigma_{2}+\sigma_{3}\right)\tau_{2}^{2m}+a\left(n+1\right)\tau_{2}^{n}-\beta }d\tau_{2},$$

and

(48) $$\Gamma =\tau_{2}\left(-\left(\tau_{2}^{m}\left(\tau_{2}^{m}\left(\tau_{2}^{m}\left(\tau_{2}^{m}\left(\tau_{2}^{m}\left(b_{6}\tau_{2}^{m}+b_{5}\right)+b_{4}\right)+b_{3}\right)+b_{2}\right)+b_{1}\right)\right)+\gamma +\omega \right).$$

**Generalized Temporal Evolution**

This subsection will reexamine the models discussed previously, incorporating generalized temporal evolution. Consequently, the framework model (1) is extended to

$$i{\left(\Phi^{l}\right)}_{t}+a{\left({\left|\Phi \right|}^{n}\Phi^{l}\right)}_{xx}+F\left({\left|\Phi \right|}^{2}\right)\Phi^{l}$$

$$=\alpha \frac{{\left|\Phi_{x}\right|}^{2}}{{\left(\Phi^{l}\right)}^{*}}+\frac{\beta }{4{\left|\Phi \right|}^{2}{\left(\Phi^{l}\right)}^{*}}\left[2{\left|\Phi \right|}^{2}{\left({\left|\Phi \right|}^{2}\right)}_{xx}-{\left\{{\left({\left|\Phi \right|}^{2}\right)}_{x}\right\}}^{2}\right]+\gamma \Phi^{l}+i\left[\lambda {\left({\left|\Phi \right|}^{2m}\Phi^{l}\right)}_{x}+\theta_{1}{\left({\left|\Phi \right|}^{2m}\right)}_{x}\Phi^{l}+\theta_{2}{\left|\Phi \right|}^{2m}{\left(\Phi^{l}\right)}_{x}\right]$$

(49) $$+\sigma_{1}{\left({\left|\Phi \right|}^{2m}\Phi^{l}\right)}_{xx}+\sigma_{2}{\left|\Phi \right|}^{2m}{\left(\Phi^{l}\right)}_{xx}+\sigma_{3}{\left|\Phi \right|}^{2m}{\left(\Phi^{l}\right)}_{xx}^{*},$$

The parameter l denotes the generalized temporal evolution parameter. As l approaches unity, the governing model reduces to (1).

The generalized temporal-evolution model (49) contains the derivative nonlinearity

$\frac{|\Phi_{x}{|}^{2}}{{\left(\Phi^{l}\right)}^{*}}=\frac{|\Phi_{x}{|}^{2}}{{\left(\Phi^{*}\right)}^{l}}.$(g)

This term is introduced as a compact gradient-dependent correction that is phase-consistent with the envelope field. Since $|\Phi_{x}{|}^{2}$ is real and measures the local steepness (gradient energy) of the profile, (g) activates only for strongly varying pulses and vanishes for spatially uniform states.

Moreover, for Φ≠0 one may rewrite (g) in the equivalent intensity-weighted form

$\frac{1}{{\left(\Phi^{*}\right)}^{l}}=\frac{\Phi^{l}}{|\Phi {|}^{2l}}\;\Rightarrow \;\alpha \;\frac{|\Phi_{x}{|}^{2}}{{\left(\Phi^{*}\right)}^{l}}=\alpha \;\frac{|\Phi_{x}{|}^{2}}{|\Phi {|}^{2l}}\;\Phi^{l},$(h) which makes explicit that this contribution is a steepness-weighted nonlinear correction scaled by the local amplitude level $|\Phi {|}^{2l}$. The use of the complex conjugate ensures gauge covariance: under $\Phi \mapsto \Phi e^{i\theta_{0}}$, the factor $1/{\left(\Phi^{*}\right)}^{l}$ acquires the compensating phase $e^{+il\theta_{0}}$, so the whole term transforms consistently with the rest of the envelope equation.

The form (g) requires Φ≠0. In this work we restrict to bright-soliton profiles with |Φ|>0 on the domain; for numerical robustness one may use the regularized implementation $\alpha \;|\Phi_{x}{|}^{2}\;\Phi^{l}/\left(|\Phi {|}^{2l}+\varepsilon \right)$ with 0<ε≪1.

In this situation, Φ(x,t) denotes the wave amplitude and is a complex-valued function. In the context of nonlinear CD, Eq. (49) does not accommodate mobile solitons unless n=0. Consequently, substituting (2) into (49), the ordinary differential equation for g(x), as stated in (3), is generalized to

$$a\left(l+n\right)g^{2l+n-2}\left(x\right)\left[g\left(x\right)g^{\prime \prime }\left(x\right)+\left(l+n-1\right){\left\{g^{\prime }\left(x\right)\right\}}^{2}\right]-\left[\gamma +l\omega -F\left\{g^{2}\left(x\right)\right\}\right]g^{2l}\left(x\right)$$

$$-g^{2l+2m-2}\left(x\right)\left[\left\{\left(l+2m\right)\left(l+2m-1\right)\sigma_{1}+l\left(l-1\right)\sigma_{2}\right\}{\left\{g^{\prime }\left(x\right)\right\}}^{2}+\left\{\left(l+2m\right)\sigma_{1}+l\sigma_{2}\right\}g\left(x\right)g^{\prime \prime }\left(x\right)\right]$$

(50) $$-l\sigma_{3}g^{2l+2m-2}\left(x\right)\left[g\left(x\right)g^{\prime \prime }\left(x\right)+\left(l-1\right){\left\{g^{\prime }\left(x\right)\right\}}^{2}\right]-\alpha {\left\{g^{\prime }\left(x\right)\right\}}^{2}-\beta g\left(x\right)g^{\prime \prime }\left(x\right)=0.$$

the imaginary component delineates the parameter restriction as:

(51) $$\left(l+2m\right)\lambda +2m\theta_{1}+l\theta_{2}=0.$$

The six forms of SPM structures proposed by Kudryashov will now be analyzed in relation to Eq. (50). The results will be presented in the following subsections.

***Form-I***

The governing model for the SPM structure represented by (6) is expressed as follows:

$$i{\left(\Phi^{l}\right)}_{t}+a{\left({\left|\Phi \right|}^{n}\Phi^{l}\right)}_{xx}+\left(\frac{b_{1}}{{\left|\Phi \right|}^{2m}}+\frac{b_{2}}{{\left|\Phi \right|}^{m}}+b_{3}{\left|\Phi \right|}^{m}+b_{4}{\left|\Phi \right|}^{2m}\right)\Phi^{l}$$

$$=\alpha \frac{{\left|\Phi_{x}\right|}^{2}}{{\left(\Phi^{l}\right)}^{*}}+\frac{\beta }{4{\left|\Phi \right|}^{2}{\left(\Phi^{l}\right)}^{*}}\left[2{\left|\Phi \right|}^{2}{\left({\left|\Phi \right|}^{2}\right)}_{xx}-{\left\{{\left({\left|\Phi \right|}^{2}\right)}_{x}\right\}}^{2}\right]+\gamma \Phi^{l}+i\left[\lambda {\left({\left|\Phi \right|}^{2m}\Phi^{l}\right)}_{x}+\theta_{1}{\left({\left|\Phi \right|}^{2m}\right)}_{x}\Phi^{l}+\theta_{2}{\left|\Phi \right|}^{2m}{\left(\Phi^{l}\right)}_{x}\right]$$

(52) $$+\sigma_{1}{\left({\left|\Phi \right|}^{2m}\Phi^{l}\right)}_{xx}+\sigma_{2}{\left|\Phi \right|}^{2m}{\left(\Phi^{l}\right)}_{xx}+\sigma_{3}{\left|\Phi \right|}^{2m}{\left(\Phi^{l}\right)}_{xx}^{*}.$$

For the SPM framework defined by (6), the ordinary differential equation g(x) is expressed as

$$a\left(l+n\right)g^{2l+n-2}\left(x\right)\left[g\left(x\right)g^{\prime \prime }\left(x\right)+\left(l+n-1\right){\left\{g^{\prime }\left(x\right)\right\}}^{2}\right]$$

$$-\left[\gamma +l\omega -\frac{b_{1}}{g^{2m}\left(x\right)}-\frac{b_{2}}{g^{m}\left(x\right)}-b_{3}g^{m}\left(x\right)-b_{4}g^{2m}\left(x\right)\right]g^{2l}\left(x\right)$$

$$-g^{2l+2m-2}\left(x\right)\left[\left\{\left(l+2m\right)\left(l+2m-1\right)\sigma_{1}+l\left(l-1\right)\sigma_{2}\right\}{\left\{g^{\prime }\left(x\right)\right\}}^{2}+\left\{\left(l+2m\right)\sigma_{1}+l\sigma_{2}\right\}g\left(x\right)g^{\prime \prime }\left(x\right)\right]$$

(53) $$-l\sigma_{3}g^{2l+2m-2}\left(x\right)\left[g\left(x\right)g^{\prime \prime }\left(x\right)+\left(l-1\right){\left\{g^{\prime }\left(x\right)\right\}}^{2}\right]-\alpha {\left\{g^{\prime }\left(x\right)\right\}}^{2}-\beta g\left(x\right)g^{\prime \prime }\left(x\right)=0.$$

Eqs. (51) and (53), when combined with the translational Lie symmetry, provide the implicit solution as shown in (10) under the constraint (14), applicable in this instance

(54) $$A=\text{exp}\left(-2\int \;\frac{\left(-\left(\left(l+2m-1\right)\left(l+2m\right)\sigma_{1}\right)-\left(l-1\right)l\left(\sigma_{2}+\sigma_{3}\right)\right)\tau_{1}^{2\left(l+m\right)}+a\left(l+n-1\right)\left(l+n\right)\tau_{1}^{2l+n}-\alpha \tau_{1}^{2}}{\tau_{1}\left(\left(\left(l+2m\right)\sigma_{1}+l\left(\sigma_{2}+\sigma_{3}\right)\right)\tau_{1}^{2\left(l+m\right)}-a\left(l+n\right)\tau_{1}^{2l+n}+\beta \tau_{1}^{2}\right)}d\tau_{1}\right),$$

(55) $$B=2\int \;\frac{\Gamma \tau_{2}^{2l-2m+1}\left(\left(\left(\left(b_{4}\tau_{2}^{m}+b_{3}\right)\tau_{2}^{m}-\gamma -l\omega \right)\tau_{2}^{m}+b_{2}\right)\tau_{2}^{m}+b_{1}\right)}{\left(\left(l+2m\right)\sigma_{1}+l\left(\sigma_{2}+\sigma_{3}\right)\right)\tau_{2}^{2\left(l+m\right)}-a\left(l+n\right)\tau_{2}^{2l+n}+\beta \tau_{2}^{2}}d\tau_{2},$$

and

(56) $$\Gamma =\text{exp}\left(-2\int \;\frac{\left(-\left(\left(l+2m-1\right)\left(l+2m\right)\sigma_{1}\right)-\left(l-1\right)l\left(\sigma_{2}+\sigma_{3}\right)\right)\tau_{1}^{2\left(l+m\right)}+a\left(l+n-1\right)\left(l+n\right)\tau_{1}^{2l+n}-\alpha \tau_{1}^{2}}{\tau_{1}\left(\left(\left(l+2m\right)\sigma_{1}+l\left(\sigma_{2}+\sigma_{3}\right)\right)\tau_{1}^{2\left(l+m\right)}-a\left(l+n\right)\tau_{1}^{2l+n}+\beta \tau_{1}^{2}\right)}d\tau_{1}\right).$$

***Form-II***

The governing model for the SPM structure, as shown in (15), is presented as follows

$$i{\left(\Phi^{l}\right)}_{t}+a{\left({\left|\Phi \right|}^{n}\Phi^{l}\right)}_{xx}+\left(\frac{b_{1}}{{\left|\Phi \right|}^{4m}}+\frac{b_{2}}{{\left|\Phi \right|}^{3m}}+\frac{b_{3}}{{\left|\Phi \right|}^{2m}}+\frac{b_{4}}{{\left|\Phi \right|}^{m}}+b_{5}{\left|\Phi \right|}^{m}+b_{6}{\left|\Phi \right|}^{2m}+b_{7}{\left|\Phi \right|}^{3m}+b_{8}{\left|\Phi \right|}^{4m}\right)\Phi^{l}$$

$$=\alpha \frac{{\left|\Phi_{x}\right|}^{2}}{{\left(\Phi^{l}\right)}^{*}}+\frac{\beta }{4{\left|\Phi \right|}^{2}{\left(\Phi^{l}\right)}^{*}}\left[2{\left|\Phi \right|}^{2}{\left({\left|\Phi \right|}^{2}\right)}_{xx}-{\left\{{\left({\left|\Phi \right|}^{2}\right)}_{x}\right\}}^{2}\right]+\gamma \Phi^{l}+i\left[\lambda {\left({\left|\Phi \right|}^{2m}\Phi^{l}\right)}_{x}+\theta_{1}{\left({\left|\Phi \right|}^{2m}\right)}_{x}\Phi^{l}+\theta_{2}{\left|\Phi \right|}^{2m}{\left(\Phi^{l}\right)}_{x}\right]$$

(57) $$+\sigma_{1}{\left({\left|\Phi \right|}^{2m}\Phi^{l}\right)}_{xx}+\sigma_{2}{\left|\Phi \right|}^{2m}{\left(\Phi^{l}\right)}_{xx}+\sigma_{3}{\left|\Phi \right|}^{2m}{\left(\Phi^{l}\right)}_{xx}^{*}.$$

The corresponding ODE for g(x) therefore is:

$$a\left(l+n\right)g^{2l+n-2}\left(x\right)\left[g\left(x\right)g^{\prime \prime }\left(x\right)+\left(l+n-1\right){\left\{g^{\prime }\left(x\right)\right\}}^{2}\right]$$

$$-\left[\gamma +l\omega -\frac{b_{1}}{g^{4m}\left(x\right)}-\frac{b_{2}}{g^{3m}\left(x\right)}-\frac{b_{3}}{g^{2m}\left(x\right)}-\frac{b_{4}}{g^{m}\left(x\right)}-b_{5}g^{m}\left(x\right)-b_{6}g^{2m}\left(x\right)-b_{7}g^{3m}\left(x\right)-b_{8}g^{4m}\left(x\right)\right]g2l\left(x\right)$$

$$-g^{2l+2m-2}\left(x\right)\left[\left\{\left(l+2m\right)\left(l+2m-1\right)\sigma_{1}+l\left(l-1\right)\sigma_{2}\right\}{\left\{g^{\prime }\left(x\right)\right\}}^{2}+\left\{\left(l+2m\right)\sigma_{1}+l\sigma_{2}\right\}g\left(x\right)g^{\prime \prime }\left(x\right)\right]$$

(58) $$-l\sigma_{3}g^{2l+2m-2}\left(x\right)\left[g\left(x\right)g^{\prime \prime }\left(x\right)+\left(l-1\right){\left\{g^{\prime }\left(x\right)\right\}}^{2}\right]-\alpha {\left\{g^{\prime }\left(x\right)\right\}}^{2}-\beta g\left(x\right)g^{\prime \prime }\left(x\right)=0.$$

Eqs. (51) and (58) combined with the translational Lie symmetry yield the implicit solution described in (10) along with the constraint in (14), where

(59) $$A=\text{exp}\left(-2\int \;\frac{\left(-\left(\left(l+2m-1\right)\left(l+2m\right)\sigma_{1}\right)-\left(l-1\right)l\left(\sigma_{2}+\sigma_{3}\right)\right)\tau_{1}^{2\left(l+m\right)}+a\left(l+n-1\right)\left(l+n\right)\tau_{1}^{2l+n}-\alpha \tau_{1}^{2}}{\tau_{1}\left(\left(\left(l+2m\right)\sigma_{1}+l\left(\sigma_{2}+\sigma_{3}\right)\right)\tau_{1}^{2\left(l+m\right)}-a\left(l+n\right)\tau_{1}^{2l+n}+\beta \tau_{1}^{2}\right)}d\tau_{1}\right),$$

(60) $$B=2\int \;\frac{\Gamma \tau_{2}^{2l-4m+1}\left(\left(\left(\left(\left(\left(\left(\left(b_{8}\tau_{2}^{m}+b_{7}\right)\tau_{2}^{m}+b_{6}\right)\tau_{2}^{m}+b_{5}\right)\tau_{2}^{m}-\gamma -l\omega \right)\tau_{2}^{m}+b_{4}\right)\tau_{2}^{m}+b_{3}\right)\tau_{2}^{m}+b_{2}\right)\tau_{2}^{m}+b_{1}\right)}{\left(\left(l+2m\right)\sigma_{1}+l\left(\sigma_{2}+\sigma_{3}\right)\right)\tau_{2}^{2\left(l+m\right)}-a\left(l+n\right)\tau_{2}^{2l+n}+\beta \tau_{2}^{2}}d\tau_{2},$$

and

(61) $$\Gamma =\text{exp}\left(-2\int \;\frac{\left(-\left(\left(l+2m-1\right)\left(l+2m\right)\sigma_{1}\right)-\left(l-1\right)l\left(\sigma_{2}+\sigma_{3}\right)\right)\tau_{1}^{2\left(l+m\right)}+a\left(l+n-1\right)\left(l+n\right)\tau_{1}^{2l+n}-\alpha \tau_{1}^{2}}{\tau_{1}\left(\left(\left(l+2m\right)\sigma_{1}+l\left(\sigma_{2}+\sigma_{3}\right)\right)\tau_{1}^{2\left(l+m\right)}-a\left(l+n\right)\tau_{1}^{2l+n}+\beta \tau_{1}^{2}\right)}d\tau_{1}\right).$$

***Form-III***

The SPM form described here is defined by Eq. (22). The governing model is

$$i{\left(\Phi^{l}\right)}_{t}+a{\left({\left|\Phi \right|}^{n}\Phi^{l}\right)}_{xx}+\left[b_{1}{\left|\Phi \right|}^{m}+b_{2}{\left|\Phi \right|}^{2m}+b_{3}{\left({\left|\Phi \right|}^{m}\right)}_{xx}\right]\Phi^{l}$$

$$=\alpha \frac{{\left|\Phi_{x}\right|}^{2}}{{\left(\Phi^{l}\right)}^{*}}+\frac{\beta }{4{\left|\Phi \right|}^{2}{\left(\Phi^{l}\right)}^{*}}\left[2{\left|\Phi \right|}^{2}{\left({\left|\Phi \right|}^{2}\right)}_{xx}-{\left\{{\left({\left|\Phi \right|}^{2}\right)}_{x}\right\}}^{2}\right]+\gamma \Phi^{l}+i\left[\lambda {\left({\left|\Phi \right|}^{2m}\Phi^{l}\right)}_{x}+\theta_{1}{\left({\left|\Phi \right|}^{2m}\right)}_{x}\Phi^{l}+\theta_{2}{\left|\Phi \right|}^{2m}{\left(\Phi^{l}\right)}_{x}\right]$$

(62) $$+\sigma_{1}{\left({\left|\Phi \right|}^{2m}\Phi^{l}\right)}_{xx}+\sigma_{2}{\left|\Phi \right|}^{2m}{\left(\Phi^{l}\right)}_{xx}+\sigma_{3}{\left|\Phi \right|}^{2m}{\left(\Phi^{l}\right)}_{xx}^{*}.$$

The governing ordinary differential equation for g(x) is expressed as

$$a\left(l+n\right)g^{2l+n-2}\left(x\right)\left[g\left(x\right)g^{\prime \prime }\left(x\right)+\left(l+n-1\right){\left\{g^{\prime }\left(x\right)\right\}}^{2}\right]-\left[\gamma +l\omega -b_{1}g^{m}\left(x\right)-b_{2}g^{2m}\left(x\right)-b_{3}{\left\{g^{m}\left(x\right)\right\}}^{{}^{\prime \prime }}\right]g^{2l}\left(x\right)$$

$$-g^{2l+2m-2}\left(x\right)\left[\left\{\left(l+2m\right)\left(l+2m-1\right)\sigma_{1}+l\left(l-1\right)\sigma_{2}\right\}{\left\{g^{\prime }\left(x\right)\right\}}^{2}+\left\{\left(l+2m\right)\sigma_{1}+l\sigma_{2}\right\}g\left(x\right)g^{\prime \prime }\left(x\right)\right]$$

(63) $$-l\sigma_{3}g^{2l+2m-2}\left(x\right)\left[g\left(x\right)g^{\prime \prime }\left(x\right)+\left(l-1\right){\left\{g^{\prime }\left(x\right)\right\}}^{2}\right]-\alpha {\left\{g^{\prime }\left(x\right)\right\}}^{2}-\beta g\left(x\right)g^{\prime \prime }\left(x\right)=0.$$

By virtue of (51), (63) and translational Lie symmetry the ODE for g(x) gives the implicit solution (10) togetehr with the constraint (14) where

(64) $$A=\text{exp}\left(-2\int \;\frac{\left(\left(-\left(\left(l+2m-1\right)\left(l+2m\right)\sigma_{1}\right)-\left(l-1\right)l\left(\sigma_{2}+\sigma_{3}\right)\right)\tau_{1}^{m}+\left(m-1\right)mb_{3}\right)\tau_{1}^{2l+m}+a\left(l+n-1\right)\left(l+n\right)\tau_{1}^{2l+n}-\alpha \tau_{1}^{2}}{\left(\left(\left(l+2m\right)\sigma_{1}+l\left(\sigma_{2}+\sigma_{3}\right)\right)\tau_{1}^{m}-mb_{3}\right)\tau_{1}^{2l+m+1}-a\left(l+n\right)\tau_{1}^{2l+n+1}+\beta \tau_{1}^{3}}d\tau_{1}\right),$$

(65) $$B=2\int \;\frac{\Gamma \tau_{2}^{2l+1}\left(\left(b_{2}\tau_{2}^{m}+b_{1}\right)\tau_{2}^{m}-\gamma -l\omega \right)}{\left(\left(\left(l+2m\right)\sigma_{1}+l\left(\sigma_{2}+\sigma_{3}\right)\right)\tau_{2}^{m}-mb_{3}\right)\tau_{2}^{2l+m}-a\left(l+n\right)\tau_{2}^{2l+n}+\beta \tau_{2}^{2}}d\tau_{2},$$

and

(66) $$\Gamma =\text{exp}\left(-2\int \;\frac{\left(\left(-\left(\left(l+2m-1\right)\left(l+2m\right)\sigma_{1}\right)-\left(l-1\right)l\left(\sigma_{2}+\sigma_{3}\right)\right)\tau_{1}^{m}+\left(m-1\right)mb_{3}\right)\tau_{1}^{2l+m}+a\left(l+n-1\right)\left(l+n\right)\tau_{1}^{2l+n}-\alpha \tau_{1}^{2}}{\left(\left(\left(l+2m\right)\sigma_{1}+l\left(\sigma_{2}+\sigma_{3}\right)\right)\tau_{1}^{m}-mb_{3}\right)\tau_{1}^{2l+m+1}-a\left(l+n\right)\tau_{1}^{2l+n+1}+\beta \tau_{1}^{3}}d\tau_{1}\right).$$

***Form-IV***

The SPM form described here is defined by Eq. (28). The governing model is

$$i{\left(\Phi^{l}\right)}_{t}+a{\left({\left|\Phi \right|}^{n}\Phi^{l}\right)}_{xx}+\left(b_{1}{\left|\Phi \right|}^{m}+b_{2}{\left|\Phi \right|}^{2m}+b_{3}{\left|\Phi \right|}^{3m}+b_{4}{\left|\Phi \right|}^{4m}+b_{5}{\left|\Phi \right|}^{5m}+b_{6}{\left|\Phi \right|}^{6m}\right)\Phi^{l}$$

$$=\alpha \frac{{\left|\Phi_{x}\right|}^{2}}{{\left(\Phi^{l}\right)}^{*}}+\frac{\beta }{4{\left|\Phi \right|}^{2}{\left(\Phi^{l}\right)}^{*}}\left[2{\left|\Phi \right|}^{2}{\left({\left|\Phi \right|}^{2}\right)}_{xx}-{\left\{{\left({\left|\Phi \right|}^{2}\right)}_{x}\right\}}^{2}\right]+\gamma \Phi^{l}+i\left[\lambda {\left({\left|\Phi \right|}^{2m}\Phi^{l}\right)}_{x}+\theta_{1}{\left({\left|\Phi \right|}^{2m}\right)}_{x}\Phi^{l}+\theta_{2}{\left|\Phi \right|}^{2m}{\left(\Phi^{l}\right)}_{x}\right]$$

(67) $$+\sigma_{1}{\left({\left|\Phi \right|}^{2m}\Phi^{l}\right)}_{xx}+\sigma_{2}{\left|\Phi \right|}^{2m}{\left(\Phi^{l}\right)}_{xx}+\sigma_{3}{\left|\Phi \right|}^{2m}{\left(\Phi^{l}\right)}_{xx}^{*}.$$

The corresponding equation for g(x) is therefore written as:

$$a\left(l+n\right)g^{2l+n-2}\left(x\right)\left[g\left(x\right)g^{\prime \prime }\left(x\right)+\left(l+n-1\right){\left\{g^{\prime }\left(x\right)\right\}}^{2}\right]$$

$$-\left[\gamma +l\omega -\frac{b_{1}}{g^{m}\left(x\right)}-b_{2}g^{2m}\left(x\right)-b_{3}g^{3m}\left(x\right)-b_{4}g^{4m}\left(x\right)-b_{5}g^{5m}\left(x\right)-b_{6}g^{6m}\left(x\right)\right]g^{2l}\left(x\right)$$

$$-g^{2l+2m-2}\left(x\right)\left[\left\{\left(l+2m\right)\left(l+2m-1\right)\sigma_{1}+l\left(l-1\right)\sigma_{2}\right\}{\left\{g^{\prime }\left(x\right)\right\}}^{2}+\left\{\left(l+2m\right)\sigma_{1}+l\sigma_{2}\right\}g\left(x\right)g^{\prime \prime }\left(x\right)\right]$$

(68) $$-l\sigma_{3}g^{2l+2m-2}\left(x\right)\left[g\left(x\right)g^{\prime \prime }\left(x\right)+\left(l-1\right){\left\{g^{\prime }\left(x\right)\right\}}^{2}\right]-\alpha {\left\{g^{\prime }\left(x\right)\right\}}^{2}-\beta g\left(x\right)g^{\prime \prime }\left(x\right)=0.$$

The translational Lie symmetry ultimately provides the implicit solution as indicated in Eq. (10), accompanied by the parameter constraint specified in Eq. (14), where

(69) $$A=\text{exp}\left(-2\int \;\frac{\left(-\left(\left(l+2m-1\right)\left(l+2m\right)\sigma_{1}\right)-\left(l-1\right)l\left(\sigma_{2}+\sigma_{3}\right)\right)\tau_{1}^{2\left(l+m\right)}+a\left(l+n-1\right)\left(l+n\right)\tau_{1}^{2l+n}-\alpha \tau_{1}^{2}}{\tau_{1}\left(\left(\left(l+2m\right)\sigma_{1}+l\left(\sigma_{2}+\sigma_{3}\right)\right)\tau_{1}^{2\left(l+m\right)}-a\left(l+n\right)\tau_{1}^{2l+n}+\beta \tau_{1}^{2}\right)}d\tau_{1}\right),$$

(70) $$B=2\int \;\frac{\Gamma \tau_{2}^{2l+1}\left(\left(\left(\left(\left(\left(b_{6}\tau_{2}^{m}+b_{5}\right)\tau_{2}^{m}+b_{4}\right)\tau_{2}^{m}+b_{3}\right)\tau_{2}^{m}+b_{2}\right)\tau_{2}^{m}+b_{1}\right)\tau_{2}^{m}-\gamma -l\omega \right)}{\left(\left(l+2m\right)\sigma_{1}+l\left(\sigma_{2}+\sigma_{3}\right),\right)\tau_{2}^{2\left(l+m\right)}-a\left(l+n\right)\tau_{2}^{2l+n}+\beta \tau_{2}^{2}}d\tau_{2}$$

where

(71) $$\Gamma =\text{exp}\left(-2\int \;\frac{\left(-\left(\left(l+2m-1\right)\left(l+2m\right)\sigma_{1}\right)-\left(l-1\right)l\left(\sigma_{2}+\sigma_{3}\right)\right)\tau_{1}^{2\left(l+m\right)}+a\left(l+n-1\right)\left(l+n\right)\tau_{1}^{2l+n}-\alpha \tau_{1}^{2}}{\tau_{1}\left(\left(\left(l+2m\right)\sigma_{1}+l\left(\sigma_{2}+\sigma_{3}\right)\right)\tau_{1}^{2\left(l+m\right)}-a\left(l+n\right)\tau_{1}^{2l+n}+\beta \tau_{1}^{2}\right)}d\tau_{1}\right).$$

***Form-V***

The SPM form described here is defined by Eq. (35). The governing model is

$$i{\left(\Phi^{l}\right)}_{t}+a{\left({\left|\Phi \right|}^{n}\Phi^{l}\right)}_{xx}+\left[b_{1}{\left|\Phi \right|}^{m}+b_{2}{\left|\Phi \right|}^{2m}+b_{3}{\left|\Phi \right|}^{3m}+b_{4}{\left|\Phi \right|}^{4m}+b_{5}{\left({\left|\Phi \right|}^{m}\right)}_{xx}+b_{6}{\left({\left|\Phi \right|}^{2m}\right)}_{xx}\right]\Phi^{l}$$

$$=\alpha \frac{{\left|\Phi_{x}\right|}^{2}}{{\left(\Phi^{l}\right)}^{*}}+\frac{\beta }{4{\left|\Phi \right|}^{2}{\left(\Phi^{l}\right)}^{*}}\left[2{\left|\Phi \right|}^{2}{\left({\left|\Phi \right|}^{2}\right)}_{xx}-{\left\{{\left({\left|\Phi \right|}^{2}\right)}_{x}\right\}}^{2}\right]+\gamma \Phi^{l}+i\left[\lambda {\left({\left|\Phi \right|}^{2m}\Phi^{l}\right)}_{x}+\theta_{1}{\left({\left|\Phi \right|}^{2m}\right)}_{x}\Phi^{l}+\theta_{2}{\left|\Phi \right|}^{2m}{\left(\Phi^{l}\right)}_{x}\right]$$

(72) $$+\sigma_{1}{\left({\left|\Phi \right|}^{2m}\Phi^{l}\right)}_{xx}+\sigma_{2}{\left|\Phi \right|}^{2m}{\left(\Phi^{l}\right)}_{xx}+\sigma_{3}{\left|\Phi \right|}^{2m}{\left(\Phi^{l}\right)}_{xx}^{*}.$$

Next, the ODE for g(x) is:

$$a\left(l+n\right)g^{2l+n-2}\left(x\right)\left[g\left(x\right)g^{\prime \prime }\left(x\right)+\left(l+n-1\right){\left\{g^{\prime }\left(x\right)\right\}}^{2}\right]$$

$$-\left[\gamma +l\omega -\frac{b_{1}}{g^{m}\left(x\right)}-b_{2}g^{2m}\left(x\right)-b_{3}g^{3m}\left(x\right)-b_{4}g^{4m}\left(x\right)-b_{5}{\left\{g^{m}\left(x\right)\right\}}^{\prime \prime }-b_{6}{\left\{g^{2m}\left(x\right)\right\}}^{\prime \prime }\right]g^{2l}\left(x\right)$$

$$-g^{2l+2m-2}\left(x\right)\left[\left\{\left(l+2m\right)\left(l+2m-1\right)\sigma_{1}+l\left(l-1\right)\sigma_{2}\right\}{\left\{g^{\prime }\left(x\right)\right\}}^{2}+\left\{\left(l+2m\right)\sigma_{1}+l\sigma_{2}\right\}g\left(x\right)g^{\prime \prime }\left(x\right)\right]$$

(73) $$-l\sigma_{3}g^{2l+2m-2}\left(x\right)\left[g\left(x\right)g^{\prime \prime }\left(x\right)+\left(l-1\right){\left\{g^{\prime }\left(x\right)\right\}}^{2}\right]-\alpha {\left\{g^{\prime }\left(x\right)\right\}}^{2}-\beta g\left(x\right)g^{\prime \prime }\left(x\right)=0.$$

This integrates, with the assistance of translational Lie symmetry, to (10) with the existence criterion specified in (14) where

(74) $$A=\text{exp}\left(-2\int \;\frac{P}{\left(\left(2m\left(\sigma_{1}-b_{6}\right)+l\left(\sigma_{1}+\sigma_{2}+\sigma_{3}\right)\right)\tau_{1}^{m}-mb_{5}\right)\tau_{1}^{2l+m+1}-a\left(l+n\right)\tau_{1}^{2l+n+1}+\beta \tau_{1}^{3}}d\tau_{1}\right),$$

(75) $$B=2\int \;\frac{\Gamma \tau_{2}^{2l+1}\left(\left(\left(\left(b_{4}\tau_{2}^{m}+b_{3}\right)\tau_{2}^{m}+b_{2}\right)\tau_{2}^{m}+b_{1}\right)\tau_{2}^{m}-\gamma -l\omega \right)}{\left(\left(2m\left(\sigma_{1}-b_{6}\right)+l\left(\sigma_{1}+\sigma_{2}+\sigma_{3}\right)\right)\tau_{2}^{m}-mb_{5}\right)\tau_{2}^{2l+m}-a\left(l+n\right)\tau_{2}^{2l+n}+\beta \tau_{2}^{2}}d\tau_{2},$$

where

(76) $$\Gamma =\text{exp}\left(-2\int \;\frac{P}{\left(\left(2m\left(\sigma_{1}-b_{6}\right)+l\left(\sigma_{1}+\sigma_{2}+\sigma_{3}\right)\right)\tau_{1}^{m}-mb_{5}\right)\tau_{1}^{2l+m+1}-a\left(l+n\right)\tau_{1}^{2l+n+1}+\beta \tau_{1}^{3}}d\tau_{1}\right),$$

and

(77) $$P=\left(\left(2m\left(2m-1\right)b_{6}-\left(l+2m-1\right)\left(l+2m\right)\sigma_{1}-\left(l-1\right)l\left(\sigma_{2}+\sigma_{3}\right)\right)\tau_{1}^{m}+\left(m-1\right)mb_{5}\right)\tau_{1}^{2l+m}+a\left(l+n-1\right)\left(l+n\right)\tau_{1}^{2l+n}-\alpha \tau_{1}^{2}.$$

***Form-VI***

From the SPM format as in (42), the governing model takes the form:

$$i{\left(\Phi^{l}\right)}_{t}+a{\left({\left|\Phi \right|}^{n}\Phi^{l}\right)}_{xx}+\left[b_{1}{\left|\Phi \right|}^{m}+b_{2}{\left|\Phi \right|}^{2m}+b_{3}{\left|\Phi \right|}^{3m}+b_{4}{\left|\Phi \right|}^{4m}+b_{5}{\left|\Phi \right|}^{5m}+b_{6}{\left|\Phi \right|}^{6m}+b_{7}{\left({\left|\Phi \right|}^{m}\right)}_{xx}+b_{8}{\left({\left|\Phi \right|}^{2m}\right)}_{xx}\right]\Phi^{l}$$

$$=\alpha \frac{{\left|\Phi_{x}\right|}^{2}}{{\left(\Phi^{l}\right)}^{*}}+\frac{\beta }{4{\left|\Phi \right|}^{2}{\left(\Phi^{l}\right)}^{*}}\left[2{\left|\Phi \right|}^{2}{\left({\left|\Phi \right|}^{2}\right)}_{xx}-{\left\{{\left({\left|\Phi \right|}^{2}\right)}_{x}\right\}}^{2}\right]+\gamma \Phi^{l}+i\left[\lambda {\left({\left|\Phi \right|}^{2m}\Phi^{l}\right)}_{x}+\theta_{1}{\left({\left|\Phi \right|}^{2m}\right)}_{x}\Phi^{l}+\theta_{2}{\left|\Phi \right|}^{2m}{\left(\Phi^{l}\right)}_{x}\right]$$

(78) $$+\sigma_{1}{\left({\left|\Phi \right|}^{2m}\Phi^{l}\right)}_{xx}+\sigma_{2}{\left|\Phi \right|}^{2m}{\left(\Phi^{l}\right)}_{xx}+\sigma_{3}{\left|\Phi \right|}^{2m}{\left(\Phi^{l}\right)}_{xx}^{*}.$$

Consequently, the ordinary differential equation for g(x) is formulated as:

$$a\left(l+n\right)g^{2l+n-2}\left(x\right)\left[g\left(x\right)g^{\prime \prime }\left(x\right)+\left(l+n-1\right){\left\{g^{\prime }\left(x\right)\right\}}^{2}\right]$$

$$-\left[\gamma +l\omega -\frac{b_{1}}{g^{m}\left(x\right)}-b_{2}g^{2m}\left(x\right)-b_{3}g^{3m}\left(x\right)-b_{4}g^{4m}\left(x\right)-b_{5}g^{5m}\left(x\right)-b_{6}g^{6m}\left(x\right)-b_{7}{\left\{g^{m}\left(x\right)\right\}}^{\prime \prime }-b_{8}{\left\{g^{2m}\left(x\right)\right\}}^{\prime \prime }\right]g^{2l}\left(x\right)$$

$$-g^{2l+2m-2}\left(x\right)\left[\left\{\left(l+2m\right)\left(l+2m-1\right)\sigma_{1}+l\left(l-1\right)\sigma_{2}\right\}{\left\{g^{\prime }\left(x\right)\right\}}^{2}+\left\{\left(l+2m\right)\sigma_{1}+l\sigma_{2}\right\}g\left(x\right)g^{\prime \prime }\left(x\right)\right]$$

(79) $$-l\sigma_{3}g^{2l+2m-2}\left(x\right)\left[g\left(x\right)g^{\prime \prime }\left(x\right)+\left(l-1\right){\left\{g^{\prime }\left(x\right)\right\}}^{2}\right]-\alpha {\left\{g^{\prime }\left(x\right)\right\}}^{2}-\beta g\left(x\right)g^{\prime \prime }\left(x\right)=0.$$

Translational Lie symmetry once again results in the implicit solution (10), with the constraint (14) ensuring its existence. Herein,

(80) $$A=\text{exp}\left(-2\int \;\frac{P\tau_{1}^{2l+m}+a\left(l+n-1\right)\left(l+n\right)\tau_{1}^{2l+n}-\alpha \tau_{1}^{2}}{\left(\left(2m\left(\sigma_{1}-b_{8}\right)+l\left(\sigma_{1}+\sigma_{2}+\sigma_{3}\right)\right)\tau_{1}^{m}-mb_{7}\right)\tau_{1}^{2l+m+1}-a\left(l+n\right)\tau_{1}^{2l+n+1}+\beta \tau_{1}^{3}}d\tau_{1}\right),$$

(81) $$B=2\int \;\frac{\Gamma \tau_{2}^{2l+1}\left(\left(\left(\left(\left(\left(b_{6}\tau_{2}^{m}+b_{5}\right)\tau_{2}^{m}+b_{4}\right)\tau_{2}^{m}+b_{3}\right)\tau_{2}^{m}+b_{2}\right)\tau_{2}^{m}+b_{1}\right)\tau_{2}^{m}-\gamma -l\omega \right)}{\left(\left(2m\left(\sigma_{1}-b_{8}\right)+l\left(\sigma_{1}+\sigma_{2}+\sigma_{3}\right)\right)\tau_{2}^{m}-mb_{7}\right)\tau_{2}^{2l+m}-a\left(l+n\right)\tau_{2}^{2l+n}+\beta \tau_{2}^{2}}d\tau_{2},$$

(82) $$\Gamma =\text{exp}\left(-2\int \;\frac{P\tau_{1}^{2l+m}+a\left(l+n-1\right)\left(l+n\right)\tau_{1}^{2l+n}-\alpha \tau_{1}^{2}}{\left(\left(2m\left(\sigma_{1}-b_{8}\right)+l\left(\sigma_{1}+\sigma_{2}+\sigma_{3}\right)\right)\tau_{1}^{m}-mb_{7}\right)\tau_{1}^{2l+m+1}-a\left(l+n\right)\tau_{1}^{2l+n+1}+\beta \tau_{1}^{3}}d\tau_{1}\right),$$

and

(83) $$P=\left(2m\left(2m-1\right)b_{8}-\left(l+2m-1\right)\left(l+2m\right)\sigma_{1}-\left(l-1\right)l\left(\sigma_{2}+\sigma_{3}\right)\right)\tau_{1}^{m}+\left(m-1\right)mb_{7}.$$

Although the present results are analytical and many profiles are given in implicit (quadrature) form, the derived existence and admissibility conditions provide actionable guidance for optical-system design. In a metamaterial waveguide, or a dispersion-engineered fiber, the coefficients entering the dispersive operator and the selected Kudryashov self-phase modulation law can be tuned through the unit-cell geometry, fill fraction, and operating frequency. The sign-compatibility requirement AB>0 and the associated reality and non-singularity conditions therefore define an existence window in parameter space where stationary localized pulses, quiescent solitons, are supported by the reduced traveling-wave dynamics. From a communications viewpoint, operating within this window reduces the likelihood of pulse spreading, radiative shedding, or distortion of the waveform, which are practical manifestations of signal degradation.

In particular, the analytical constraints can be used as a pre-screening tool before full PDE simulation or experiments. Given a candidate dispersion map and nonlinear-response model, for example Forms I to VI, one computes the reduced coefficients A and B and checks whether AB>0. If AB<0, the quiescent homoclinic branch is precluded under the present reduction, indicating that stationary localization is not supported and that a pulse launched near the targeted operating point is expected to broaden or develop a trailing structure. Conversely, when AB>0 and the admissibility conditions are satisfied, the model predicts that localized profiles exist, and parameter sweeps quantify how the Kudryashov coefficients and dispersive parameters control the pulse amplitude and width. This information can be translated into engineering bounds on operating power and dispersion strength to mitigate waveform deterioration. In signal-processing terms, the implicit profiles can be used as matched templates, as initial conditions in split-step simulations, or as benchmark shapes for pulse-shaping filters, enabling systematic exploration of how close a practical input must be to the admissible soliton family to avoid stalling or distortion during propagation.

The present framework can be extended by incorporating more general non-Hamiltonian effects relevant to optical communications, such as distributed gain and loss, filtering, saturable absorption, Raman response, or higher-order self-steepening. Mathematically, these contributions enter as additional nonconservative terms in the envelope equation and generally break the exact integrability of the reduced profile ODE. In such cases, the Lie-symmetry reduction and quadrature structure may persist only approximately, and the dynamics must be analyzed using perturbation theory for homoclinic orbits, collective-coordinate reductions, or numerical continuation of stationary profiles. Incorporating these effects would allow one to quantify how gain and loss management and filtering shift the admissible soliton window, and how robust the implicit soliton families are under realistic dissipative perturbations.

## Method validation

The Lie-symmetry-based reduction is corroborated through three complementary checks that address, respectively, the mathematical consistency of the reduction, the existence and admissibility of the resulting profiles, and the physical plausibility of the retrieved stationary structures.

First, starting from the governing partial differential model, we employ the stationary ansatz $\Phi \left(x,t\right)=g\left(x\right)e^{i\omega t}$ to obtain a consistent ordinary differential equation for g(x). This reduced ODE admits the translational Lie symmetry X=∂/∂x, which yields a first integral and allows the solution to be expressed in quadrature form.

Second, since the quadrature involves a real-valued integrand, the implicit quiescent soliton profiles are admissible only in parameter regimes where the positivity condition AB>0 holds. This requirement furnishes a practical and explicit parameter constraint ensuring the existence of physically meaningful (real) stationary profiles.

**Fig. 1:**
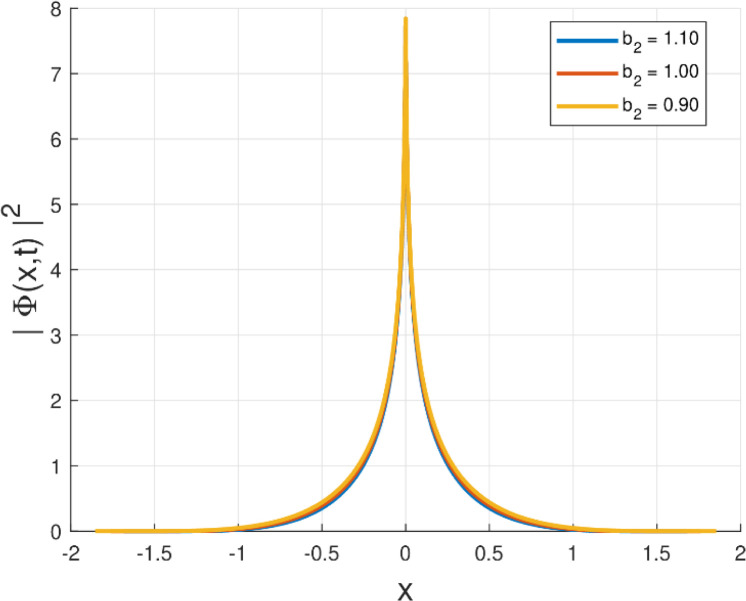
Intensity profile $|\Phi \left(x,t\right){|}^{2}$ of the quiescent soliton solution for Form I (SPM), shown for different values of the parameter $b_{2}$ ($b_{2}=1.10,\;1.00,\;0.90$) while keeping the remaining parameters fixed.

**Fig. 2:**
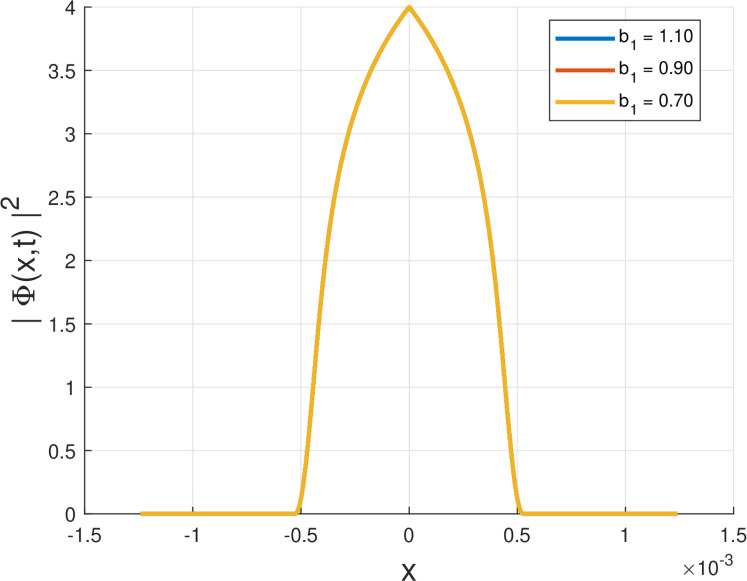
Intensity profile $|\Phi \left(x,t\right){|}^{2}$ of the quiescent soliton solution for Form II (SPM), shown for different values of the parameter $b_{1}$ ($b_{1}=1.10,\;0.90,\;0.70$) while keeping the remaining parameters fixed.

**Fig. 3:**
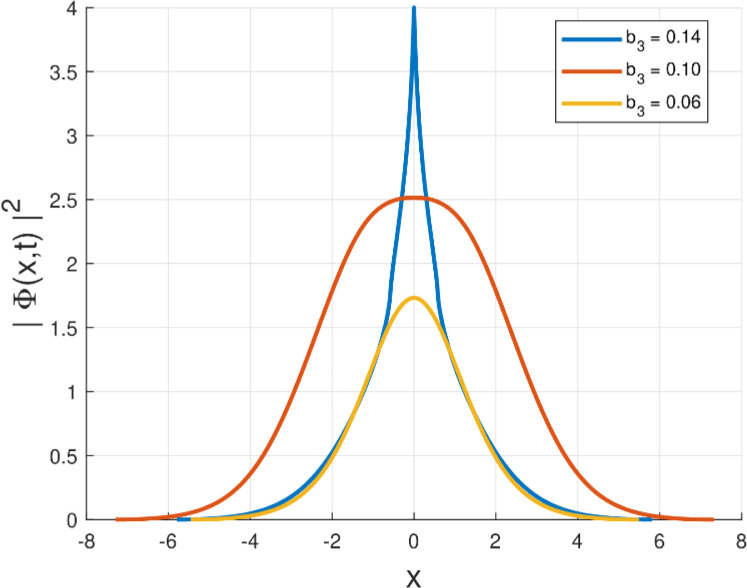
Intensity profile $|\Phi \left(x,t\right){|}^{2}$ of the quiescent soliton solution for Form III (SPM), shown for different values of the parameter $b_{3}$ ($b_{3}=0.14,\;0.10,\;0.06$) while keeping the remaining parameters fixed.

**Fig. 4:**
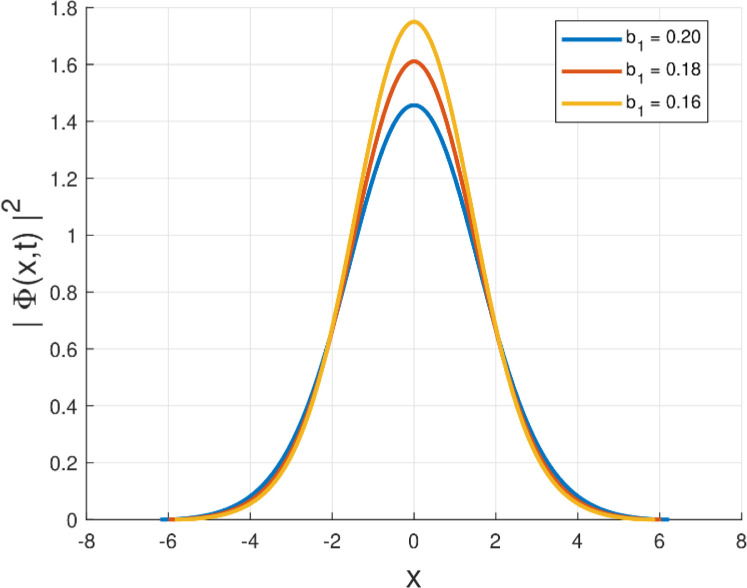
Intensity profile $|\Phi \left(x,t\right){|}^{2}$ of the quiescent soliton solution for Form IV (SPM), shown for different values of the parameter $b_{1}$ ($b_{1}=0.20,\;0.18,\;0.16$) while keeping the remaining parameters fixed.

**Fig. 5:**
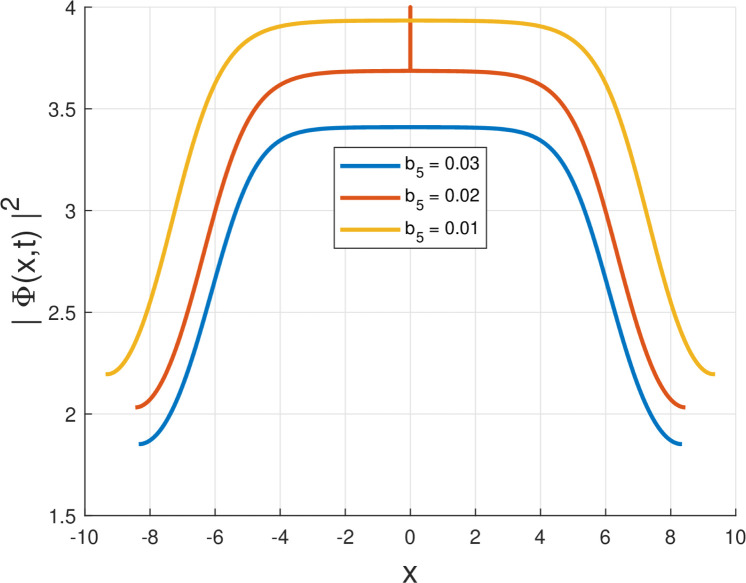
Intensity profile $|\Phi \left(x,t\right){|}^{2}$ of the quiescent soliton solution for Form V (SPM), shown for different values of the parameter $b_{5}$ ($b_{5}=0.03,\;0.02,\;0.01$) while keeping the remaining parameters fixed.

**Fig. 6:**
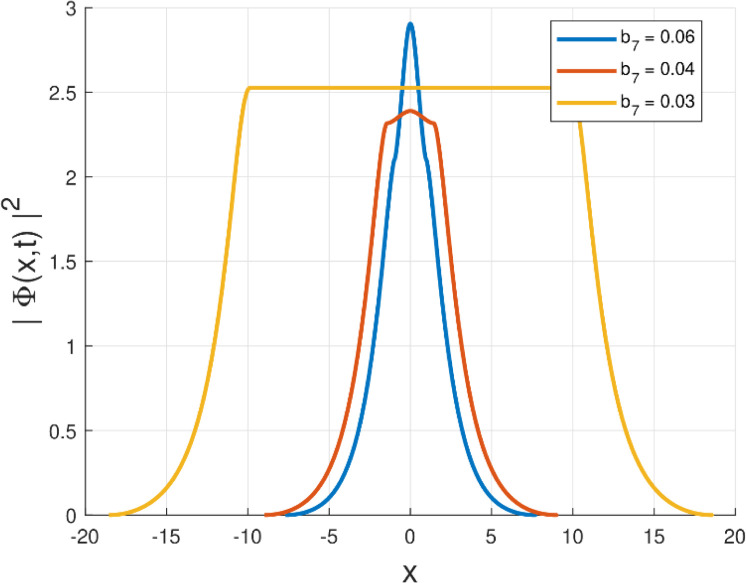
Intensity profile $|\Phi \left(x,t\right){|}^{2}$ of the quiescent soliton solution for Form VI (SPM), shown for different values of the parameter $b_{7}$ ($b_{7}=0.06,\;0.04,\;0.03$) while keeping the remaining parameters fixed.

Third, the physical relevance of the obtained solutions is illustrated by plotting the intensity $|\Phi \left(x,t\right){|}^{2}=|g\left(x\right){|}^{2}$ for the Kudryashov self-phase modulation structures (Forms I–VI). In particular, Fig. 1, Fig. 2, Fig. 3, Fig. 4, Fig. 5, Fig. 6 demonstrate localized stationary patterns and show how representative SPM parameters (e.g., $b_{1},b_{3},b_{5}$) modulate the amplitude and width while preserving localization.

An analogous validation applies to the generalized temporal evolution model: the same reduction mechanism produces the corresponding ODE, the translational symmetry again leads to a quadrature representation, and the admissible parameter regime is characterized by the associated constraint inherited from the imaginary part of the reduced system.

To enable direct verification of the soliton profiles and their parameter dependence, the numerical intensity profiles corresponding to Figs. (1) – (6) are included below (and also provided as separate high-resolution figure files in the submission package).

## Limitations

The present analysis provides implicit (quadrature-based) quiescent soliton profiles; explicit closed-form expressions are generally not available. The parameter constraints ensuring existence (e.g., positivity conditions) may restrict admissible regimes for certain material settings.

The present study derived quiescent optical solitons from the CGLE, including nonlinear CD and various kinds of Kudryashov’s SPM structure. Lie symmetry analysis is the mathematical methodology used to retrieve these solitons. The findings are expressed in terms of quadratures and are hence non-local. Thus, the title of this study. The findings of this article convey an important message to the optoelectronics community.

It is essential to ensure that the CD does not inadvertently become nonlinear during soliton transmission over subterranean or underwater cables across intercontinental distances. This applies to all types of optoelectronic devices used in the telecommunications sector, including optical fibers, optical couplers, magneto-optic waveguides, crystals, optical metamaterials, and dispersion-flattened fibers, among others. The outcomes would be disastrous with impassive solitons, as seen and represented. Consequently, communications engineers must exert heightened diligence to mitigate this fallout. New techniques in signal processing and adaptive modulation are being explored to enhance the stability of soliton transmission. By implementing advanced algorithms that can dynamically adjust to varying conditions, engineers aim to maintain the integrity of the transmitted signals across these challenging mediums.

Although the analysis is carried out at the level of a reduced traveling-wave dynamical system, the resulting admissibility conditions can be translated into practical *design-side parameter screens* for optical links and metamaterial waveguides. In particular, the inequality AB>0 is a compact way of enforcing that the reduced profile ODE admits a real solitary-wave branch (homoclinic orbit) and that the corresponding intensity profile is bounded and localized. From an engineering viewpoint, this amounts to a *sign compatibility* requirement between the effective dispersive response and the effective nonlinear response: only parameter sets satisfying AB>0 support stationary localized pulses in the present model class.

Concretely, the workflow for system design is as follows.

(i) Choose the intended SPM law (Form I–VI) and material operating point (carrier frequency, effective dispersion and loss/gain settings), which fixes the physical coefficients that enter A and B.
(ii) Compute A and B from their explicit formulas and retain only those parameter combinations for which AB>0; this provides a first-pass *existence window* for non-dispersive localized transmission.
(iii) If AB<0, the reduced ODE has no real homoclinic branch under the quiescent ansatz, so the model predicts that stationary localized pulses cannot persist and will instead broaden, radiate, or undergo modulation, which corresponds to signal degradation in propagation.

To make this screening criterion directly usable, we report representative admissible and inadmissible parameter sets for one SPM form and indicate how the inequality restricts a design parameter (e.g., a nonlinear coefficient $b_{j}$ or an effective dispersion coefficient). The resulting bounds can be interpreted as engineering limits on the accessible operating regime: within the AB>0 window, localized pulse transmission is supported by the model, whereas outside this window one should expect deterioration of pulse integrity.

The implicit quadratures derived here provide a unified representation of quiescent soliton families, but their practical use requires numerical evaluation. Two challenges are most common. First, the integrands may contain turning points and weak singularities near equilibrium roots of the reduced polynomial, which requires careful treatment to maintain accuracy. In practice, one evaluates the profile by locating the relevant roots that define the admissible interval, performing a change of variables that removes the square-root singular behavior near simple roots, and using adaptive quadrature with prescribed absolute and relative tolerances. Second, parameter regimes close to the boundary of admissibility, for example $AB\to 0^{+}$ or regimes near multiple roots, are numerically stiff. Small changes in parameters can cause large changes in the profile width, and the quadrature becomes ill-conditioned. For reproducibility, we therefore recommend reporting the root structure, the integration interval, and the numerical tolerances alongside any plotted profiles.

A further limitation is that our existence conditions are derived for quiescent reductions of the governing equation and should be interpreted as necessary conditions for stationary localization under that ansatz. A full PDE stability analysis, including spectral stability, modulational instability, and noise sensitivity, is beyond the scope of the present work and will be addressed separately.

## Ethics statements

This work does not involve human subjects or animal experiments. There are no ethical concerns related to this study.

## CRediT author statement

Abdullahi Rashid Adem: Methodology, Software, Formal analysis, Writing – Original Draft. Ahmed H. Arnous: Validation, Visualization, Data curation, Writing – Review & Editing. Houria Triki: Investigation, Resources, Writing – Review & Editing. Oswaldo González–Gaxiola: Data curation, Formal analysis, Visualization, Writing – Review & Editing. Lina S. Calucag: Investigation, Resources, Writing – Review & Editing. Anjan Biswas: Conceptualization, Methodology, Supervision, Funding acquisition, Writing – Review & Editing.

## Declaration of interests

The authors declare that they have no known competing financial interests or personal relationships that could have appeared to influence the work reported in this paper.

## Data Availability

No data was used for the research described in the article.

## References

[1] Adem A.R., Yildirim Y., Moraru L., González–Gaxiola O., Biswas A. (2025). Implicit quiescent optical soliton perturbation having nonlinear chromatic dispersion and generalized temporal evolution with Kudryashov’s forms of self-phase modulation structure by Lie symmetry. Afrika Matematica.

[2] Biswas A., Ekici M., Sonmezoglu A., Belic M. (2018). Stationary optical solitons with nonlinear group velocity dispersion by extended trial function scheme. Optik. (Stuttg).

[3] Arnous A.H., Biswas A., Yildirim Y., Moraru L., Moldovanu S., Iticescu C., Khan S., Alshehri H.M. (2023). Quiescent optical solitons with quadratic–cubic and generalized quadratic–cubic nonlinearities. Telecom.

[4] Ekici M. (2022). Stationary optical solitons with complex Ginzburg–Landau equation having nonlinear chromatic dispersion and Kudryashov’s refractive index structures". Phys. Lett. A.

[5] Ekici M. (2022). Kinky breathers, W–shaped and multi–peak soliton interactions for Kudryashov’s quintuple power–law coupled with dual form of non–local refractive index structure. Chaos, Solit. Fract..

[6] Ekici M. (2022). Optical solitons with Kudryashov’s quintuple power–law coupled with dual form of non–local law of refractive index with extended Jacobi’s elliptic function". Opt. Quantum. Electron..

[7] Ekici M. (2023). Stationary optical solitons with Kudryashov’s quintuple power law nonlinearity by extended Jacobi’s elliptic function expansion. J. Nonlinear Opt. Phys. Mater..

[8] Arnous A.H., Moraru L. (2022). Optical solitons with the complex Ginzburg–Landau equation with Kudryashov’s law of refractive index. Mathematics.

[9] Kudryashov N.A. (2022). Stationary solitons of the generalized nonlinear Schrödinger equation with nonlinear dispersion and arbitrary refracttive index. Appl. Math. Lett..

[10] Kudryashov N.A. (2024). Stationary solitons of the model with nonlinear chromatic dispersion and arbitrary refractive index. Optik. (Stuttg).

[11] Jawad A.J.M., Abu–AlShaeer M.J. (2023). Highly dispersive optical solitons with cubic law and cubic–quintic–septic law nonlinearities by two methods". Al–Rafidain J. Eng. Sci..

[12] Jihad N., Almuhsan M.A.A. (2023). Evaluation of impairment mitigations for optical fiber communications using dispersion compensation techniques. Al–Rafidain J. Eng. Sci..

[13] Smertenko P., Maksimenko Z., Belyaev A. (2025). Quantum Innovations and the SPQEO journal. Semicond. Phys., Quant. Electron. Optoelectron..

[14] Tiofack C.G.L., Mohamadou A., Alim (2012). Modulational instability in metamaterials with saturable nonlinearity and higher–order dispersion". J. Mod. Opt..

[15] Yalci A.M., Ekici M. (2022). Stationary optical solitons with complex Ginzburg-Landau equation having nonlinear chromatic dispersion. Opt. Quantum. Electron..

[16] Yan Z. (2006). Envelope compact and solitary pattern structures for the GNLS(m,n,p,q) equations. Phys. Lett. A.

[17] Han T., Li Z., Li C., Zhao L. (2023). Bifurcations, stationary optical solitons and exact solutions for complex Ginzburg–Landau equation with nonlinear chromatic dispersion in non–Kerr law media. J. Opt..

[18] L. Tang & H. Zeng. “The study of optical solitons and traveling wave solutions for the Lakshmanan-Porsezian-Daniel system with Kerr law of nonlinear refractive index". To appear in *J. Opt.*. DOI: 10.1007/s12596-025-02689-5.

[19] L. Kaur & A.-.-.M. Wazwaz. “Gaussons for generalized nonlinear Schrödinger equation equipped with logarithmic nonlinearity and variable coefficients, depicting the propagation of optical pulses". To appear in *J. Opt.*. DOI: 10.1007/s12596-025-02572-3.

[20] Kasapeteva Z. (2025). Energy exchange between the polarization components of an optical pulse under the influence of degenerate four–photon parametric processes". Trans. Opt. Photon..

